# Strain-induced bandgap engineering in 2D ψ-graphene materials: a first-principles study

**DOI:** 10.3762/bjnano.15.116

**Published:** 2024-11-20

**Authors:** Kamal Kumar, Nora H de Leeuw, Jost Adam, Abhishek Kumar Mishra

**Affiliations:** 1 Department of Physics, Applied Science Cluster, School of Advanced Engineering, University of Petroleum and Energy Studies (UPES), Bidholi via Premnagar, Dehradun, Uttarakhand 248007, India; 2 School of Chemistry, University of Leeds, Leeds LS2 9JT, UK; 3 Department of Earth Sciences, Utrecht University, 3584 CB Utrecht, Netherlands; 4 Computational Materials and Photonics, Electrical Engineering and Computer Science (FB 16) and Institue of Physics (FB 10), University of Kassel, Wilhelmshöher Allee 71, 34121 Kassel, Germany; 5 Center for Interdisciplinary Nanostructure Science and Technology, University of Kassel, Heinrich-Plett-Straße 40, 34132 Kassel, Germany

**Keywords:** 2D materials, defects, DFT, graphene, ψ-graphene, strain

## Abstract

High mechanical strength, excellent thermal and electrical conductivity, and tunable properties make two-dimensional (2D) materials attractive for various applications. However, the metallic nature of these materials restricts their applications in specific domains. Strain engineering is a versatile technique to tailor the distribution of energy levels, including bandgap opening between the energy bands. ψ-Graphene is a newly predicted 2D nanosheet of carbon atoms arranged in 5,6,7-membered rings. The half and fully hydrogenated (hydrogen-functionalized) forms of ψ-graphene are called ψ-graphone and ψ-graphane. Like ψ-graphene, ψ-graphone has a zero bandgap, but ψ-graphane is a wide-bandgap semiconductor. In this study, we have applied in-plane and out-of-plane biaxial strain on pristine and hydrogenated ψ-graphene. We have obtained a bandgap opening (200 meV) in ψ-graphene at 14% in-plane strain, while ψ-graphone loses its zero-bandgap nature at very low values of applied strain (both +1% and −1%). In contrast, fully hydrogenated ψ-graphene remains unchanged under the influence of mechanical strain, preserving its initial characteristic of having a direct bandgap. This behavior offers opportunities for these materials in various vital applications in photodetectors, solar cells, LEDs, pressure and strain sensors, energy storage, and quantum computing. The mechanical strain tolerance of pristine and fully hydrogenated ψ-graphene is observed to be −17% to +17%, while for ψ-graphone, it lies within the strain span of −16% to +16%.

## Introduction

Graphene is the best-known zero-bandgap two-dimensional (2D) material, consisting of a single layer of sp^2^-hybridized carbon atoms arranged together in a hexagonal lattice [1]. Because of its extraordinary electrical and thermal conductivity, large surface area, and easy chemical functionalization, it provides a variety of applications in pliable displays and as strengthening material in composites [2–4]. It has also gained considerable attention among researchers for its application in hydrogen storage, owing to its good adsorption capacity and controllable storage and re-release of hydrogen at efficient temperatures [4–5]. The geometrical structures of graphene obtained from its half and full hydrogenation are called, respectively, graphone [6] and graphane [7]. Zhao et al. have reported the successful synthesis of graphone on a Ni(111) surface [8]. Their X-ray photoelectron diffraction (XPD), temperature programmed desorption (TPD), and density functional theory (DFT) study suggests that the hydrogenation of graphene with atomic hydrogen leads to the formation of graphone [8]. The full hydrogenation of graphene (graphane) was experimentally obtained by Elias et al., and their TEM and Raman spectroscopy results evidence the transition of graphene from a semimetal to an insulator after full hydrogenation [9]. After the discovery of graphene, other novel 2D materials, such as goldene [10], stanene [11], plumbene [12], antimonene [13], and arsenene [14], have been predicted and experimentally synthesized. Few of these materials are zero-bandgap, like goldene [15] and ψ-graphene [16]. The absence of bandgaps in 2D materials makes them unsuitable for conventional semiconductor applications and limits their use in photonics and optical devices [17]. Therefore, bandgap engineering (manipulation of electronic band structures (EBSs)) of these materials becomes essential to expand their utility in energy-related and optoelectronic applications [18–19]. Engineering of the electronic gap not only broadens the possible use of 2D materials but also enables them to satisfy the demand for ultramodern technologies [20]. Bandgap engineering can be achieved through different techniques like (i) doping, where the introduction of dopants or impurities modifies the EBS [21], (ii) strain engineering by inserting mechanical strain to alter the electronic properties [22–23], and (iii) defect engineering [24]. Among these techniques, strain engineering is an advantageous method because of its versatility (it can be used for a wide range of materials) [25], precise control (it can efficiently increase and decrease the bandgap according to requirements) [26], non-destructive nature (intrinsic properties of the materials can be preserved) [27], and compatibility with established technologies (the semiconductor industry can adopt it to enhance the performance of devices) [28]. Strain can be introduced in graphene using different methods, namely, by exploiting a mismatch in thermal expansion between graphene and the underlying substrate, by transferring graphene to a piezoelectric substrate, by shrinking or elongating the substrate by applying a bias voltage, or by using the tip of an atomic force microscope (AFM) to push graphene over a hole created in the substrate [29]. A wealth of literature on strain engineering of graphene and other 2D materials using different experimental techniques is available. Ni et al. synthesized graphene on a polyethylene terephthalate (PET) substrate and studied the effect of uniaxial strain through Raman spectroscopy [30]. They stretched PET in one direction and found a redshift in the D and G bands for a single graphene layer. Also, uniaxial strain of 0.8% can be introduced in graphene by stretching [30]. Conley et al. studied the effect of uniaxial tensile strain on mono- and bilayer MoS_2_, where the strain was introduced in MoS_2_ through a four-point bending apparatus and a transition from an optical direct bandgap to an optical indirect bandgap in MoS_2_ at 1% strain was observed [31].

Gui et al. examined the EBS of graphene exposed to different planar strain patterns using both first-principles and tight-binding approaches [32]. They found that graphene maintains its zero-bandgap nature under the influence of symmetrical strain [32]. However, when it underwent asymmetrical strain, the bandgap reached 0.486 eV (on applying strain parallel to the C–C bonds) and 0.170 eV (on applying strain perpendicular to the C–C bonds) at 12.2% and 7.3% strain, respectively [32]. Kerszberg et al. have used density functional theory (DFT) calculations to investigate the modification of the electronic properties of graphene through strain engineering [33]. They found that isotropic and biaxial strains cannot open graphene’s bandgap [33]. In contrast, the presence of biaxial strain and compression along zig-zag (11%) and armchair (−20%) directions can open the bandgap of graphene up to 1 eV [33]. The application of strain engineering is not restricted to tailoring the electronic properties of graphene; it can also be employed to change the electronic characteristics of novel 2D post-graphene materials [34–36]. Xu et al. found a shift from an indirect bandgap to a direct bandgap in arsenene under uniaxial strain along the zig-zag direction [37]. Mohan et al. employed DFT to study the effect of strain on the electrical band structure of a silicene monolayer and found a bandgap (335 meV) opening in silicene at 4% compressive uniaxial strain [34]. At 6% strain, a maximum bandgap of 389 meV and 379 meV was observed for uniaxial and biaxial strains, respectively. When the applied strain exceeds a threshold of 8%, the bandgap of silicene disappears [34].

In 2017, Li et al. predicted a new 2D allotrope of carbon atoms, using first-principles calculations, named ψ-graphene [16]. It is a flat sheet of 5,6,7-membered carbon rings that is dynamically and thermally stable [16,38]. It can be constructed from the short-chain hydrocarbon *s*-indacene and has the chemical formula C_12_H_8_ [16]. Because of the absence of a bandgap, ψ-graphene can be used in optical detectors [39]. However, a first principles-based computational study has shown that its zero bandgap is a major challenge to its suitability in optoelectronic and electronic devices [40]. Despite being less stable than graphene, the mechanical properties of ψ-graphene are similar, and on increasing the ratio of hexagonal rings in ψ-graphene (i.e., *n*-hex-ψ-graphene), its total energy was found to be −9.23 eV/atom, which is identical to pristine graphene [16]. Another effective method to enhance the stability of ψ-graphene is edge hydrogenation, which leads to a metal-to-insulator transition, making it suitable for operating at ultrahigh speeds. ψ-Graphone and ψ-graphane are, respectively, the half and fully hydrogenated forms of ψ-graphene [41]. Like graphene, ψ-graphone possesses a zero bandgap, but ψ-graphane is an insulator with a bandgap of 4.13 eV [39]. Although a successful experimental synthesis of ψ-graphene has not yet been realized, many theoretical investigations have been carried out by different research teams to study its various potential applications in sensors, lithium-ion batteries, and hydrogen storage [16,39,42]. We have recently employed detailed density functional theory calculations with dispersion correction and on-site Coulomb interaction (DFT(D) + U) to investigate CO_2_ activation on ψ-graphene and its hydrogenated forms for their application in the electrochemical conversion of CO_2_ [43]. Faghihnasiri et al. have performed DFT calculations and concluded that ψ-graphene has the potential to be employed in infrared (IR) sensors, ultraviolet (UV) optomechanical sensors, and visible-light sensors [39]. Li et al. theoretically reported a maximum theoretical storage capacity of 372 mAh·g^−1^ for Li, showing its capability to be utilized as an anode material in Li-ion batteries [16]. Theoretical investigations also suggest that when ψ-graphene is decorated with transition metals like zirconium, yttrium, and titanium, it can serve as an excellent adsorbent for hydrogen storage [42,44–45]. DFT calculations have shown that the adsorption energies of hydrogen molecules over Zr-, Y-, and Ti-decorated ψ-graphene are found to lie within the standard range of −0.2 to −0.7 eV specified by the Department of Energy (DoE) [42,44–45]. However, bandgap engineering, for example, passivation, doping, or strain engineering, is crucial to modify and improve the bandgap for various electronic applications. In this study, we have investigated the electronic properties of pristine and hydrogenated ψ-graphene (i.e., ψ-graphone and ψ-graphane) under the influence of uniform biaxial mechanical strain (positive and negative).

## Results and Discussion

### Structural and electronic properties of 2D nanosheets without strain

**Figure 1:**
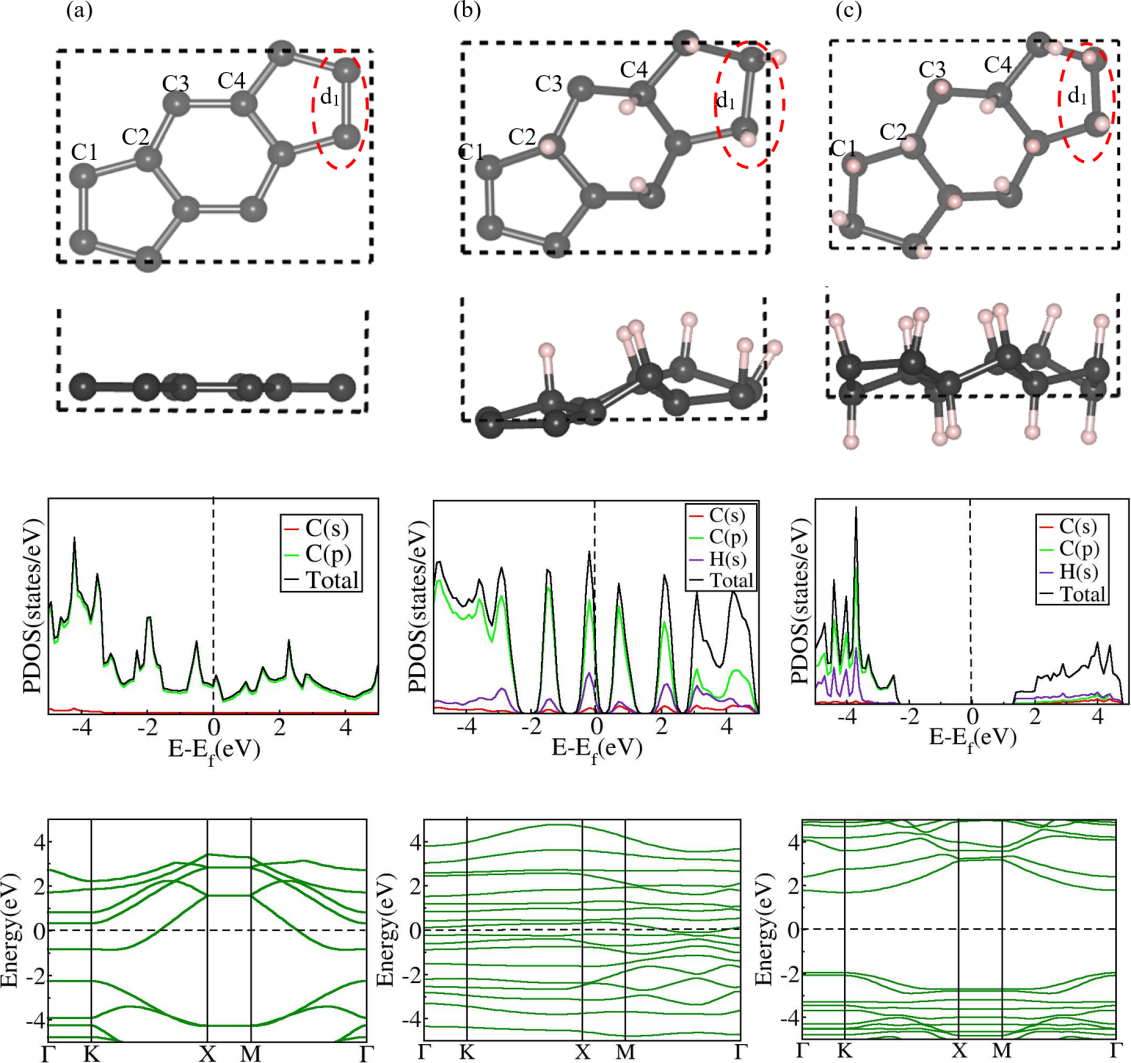
Top and side views of relaxed geometries, PDOS, and EBSs of (a) ψ-graphene, (b) ψ-graphone, and (c) ψ-graphane. The black dashed square boxes in the structures represent the unit cells. Black and pink balls are carbon and hydrogen atoms, respectively. Both ψ-graphene and ψ-graphone exhibit zero bandgaps, while ψ-graphane has a wide bandgap of 3.78 eV.

We first estimated lattice parameters, bond lengths, and atomic positions of our 2D nanosheets (ψ-graphene, ψ-graphone, and ψ-graphone) by utilizing structural details available from the literature [38–39]. Subsequently, we have relaxed the nanosheets using the established computational parameters outlined in the Computational Methodology section. Table 1 summarizes the relaxed cell lattice parameters and C–C and C–H bond lengths of the three materials, which match well with earlier DFT results [38–39]. Each nanosheet has four inequivalent carbon atoms labeled C1, C2, C3, and C4, possessing unique chemical and physical attributes (Figure 1). At the junction of three *s*-indacene molecules, the C–C bond length *d*_1_ is significantly longer than typical C–C bonds (Figure 1).

**Table 1:** Optimized lattice parameters (*a*, *b*), carbon–carbon bond lengths (*d*_1_, *d*_C–C_), carbon–hydrogen bond lengths (*d*_C–H_), buckling heights (*h*), and bandgap energies (*E*_g_) of ψ-graphene, ψ-graphone, and ψ-graphane 2D nanosheets.

Material		*a* (Å)	*b* (Å)	*d*_1_ (Å)	*d*_C–C_ (Å)	*d*_C–H_ (Å)	*h* (Å)	*E*_g_ (eV)

ψ-graphene	our work	6.70	4.83	1.51	1.42–1.44	—	0.00	zero
	previous work [38]	6.70	4.84	1.51	1.41–1.44	—	0.00	zero
ψ-graphone	our work	6.70	4.83	1.61	1.47–1.57	1.14	1.79	zero
	previous work [39]	6.70	4.84	—	—	—	—	zero
ψ-graphane	our work	6.71	4.83	1.56	1.49–1.55	1.11	0.85	3.78
	previous work [41]	6.70	4.84	1.53	1.52–1.53	—	—	4.13

To investigate the electronic properties of these 2D nanosheets, we performed EBS and density of states (DOS) calculations (Figure 1) alongside projected orbital calculations of the different atoms to understand their contributions to the electronic states. Our calculations reveal no gap between the energy bands in both ψ-graphene and ψ-graphone (Figure 1a,b), with finite states present at the Fermi level (*E*_f_). The dominant energy orbitals in the projected density of states (PDOS) of ψ-graphene and ψ-graphone are the outermost C 2p orbitals. In the fully hydrogenated form, that is, in ψ-graphane, we note a discernible separation of 3.78 eV between the valence and conduction bands in the EBS. This material is a direct-bandgap material with the alignment of the conduction band’s minima and valence band’s maxima at the same k-points in the Brillouin zone (Figure 1c). We tabulate the structural parameters, bandgap, and buckling heights of these structures in Table 1. From Table 1 and Figure 1, it is clear that, while ψ-graphene is a flat 2D material, ψ-graphone and ψ-graphane are buckled sheets, and their buckling heights are 1.79 and 0.85 Å, respectively.

### Structural and electronic properties of ψ-graphene with strain

We tabulate the structural parameters, buckling heights, and electronic bandgap values of all strained structures in Table 2.

**Table 2:** Lattice parameters (*a*, *b*), C–C bond lengths (*d*_1_, *d*_C–C_), average C–C bond length (*d*_C–C_(avg)), buckling height (*h*), and bandgap energy (*E*_g_) of ψ-graphene on applying lattice strain.

Applied strain	Lattice parameters (Å)		*d* (Å)			*h* (Å)	*E*_g_ (eV)	Bandgap type
	
	*a*	*b*	*d*_1_	*d*_C–C_	*d*_C–C_(avg)			

−17%	5.56	4.01	1.36	1.37–1.59	1.48	1.956	zero	—
−16%	5.63	4.06	1.34	1.32–1.53	1.42	2.232	0.700	indirect
−15%	5.69	4.11	1.35	1.32–1.53	1.42	2.182	0.400	indirect
−14%	5.76	4.15	1.36	1.32–1.52	1.42	2.132	0.200	direct
−13%	5.83	4.21	1.35	1.34–1.44	1.39	1.903	zero	—
−10%	6.03	4.35	1.27	1.34–1.33	1.34	0.007	zero	—
−7%	6.23	4.50	1.38	1.31–1.34	1.32	0.005	zero	—
−5%	6.36	4.59	1.41	1.34–1.37	1.35	0.004	zero	—
−3%	6.50	4.69	1.45	1.36–1.40	1.38	0.004	zero	—
−2%	6.57	4.74	1.47	1.38–1.41	1.40	0.004	zero	—
−1%	6.63	4.01	1.49	1.39–1.42	1.41	0.004	zero	—
0%	6.70	4.83	1.51	1.42–1.44	1.42	—	zero	—
1%	6.77	4.88	1.49	1.40–1.42	1.41	0.004	zero	—
2%	6.83	4.93	1.49	1.39–1.42	1.41	0.004	zero	—
3%	6.90	4.98	1.49	1.39–1.42	1.41	0.004	zero	—
5%	7.03	5.08	1.49	1.39–1.42	1.41	0.004	zero	—
7%	7.17	5.17	1.49	1.39–1.40	1.40	0.004	zero	—
10%	7.37	5.46	1.49	1.39–1.40	1.40	0.004	zero	—
13%	7.57	5.46	1.49	1.39–1.42	1.41	0.004	zero	—
14%	7.64	5.51	1.49	1.39–1.42	1.41	0.004	zero	—
15%	7.71	5.56	1.49	1.39–1.42	1.41	0.004	zero	—
16%	7.77	5.61	1.49	1.39–1.42	1.41	0.004	zero	—
17%	7.84	5.66	1.49	1.39–1.42	1.41	0.004	zero	—

#### Positive strain

We applied positive strain toward deliberate expansion of the structure, particularly focusing on the lattice plane, varying its value from 1% to 17% (Supporting Information File 1, Figure S1). We observed that the positive strain fails to open the bandgap in ψ-graphene (Table 2). To comprehensively analyze the impact of this positive strain on the electrical properties of ψ-graphene, we have also plotted the PDOSs and the EBSs of all the strained structures of ψ-graphene in Figure S2 and Figure S3 (Supporting Information File 1), respectively. We observed that on applying positive strain, *d*_1_, a C–C bond length in ψ-graphene changes to 1.49 Å in all strained structures from its initial value of 1.51 Å. Concurrently, the remaining C–C bond lengths remain uniform with a mean value of 1.40–1.41 Å, as shown in Table 2. Moreover, the ψ-graphene nanosheet remains almost flat, with a buckling height of only 0.004 Å, indicating the stability of the remaining C–C bonds even under the influence of positive strain.

#### Negative strain

**Figure 2:**
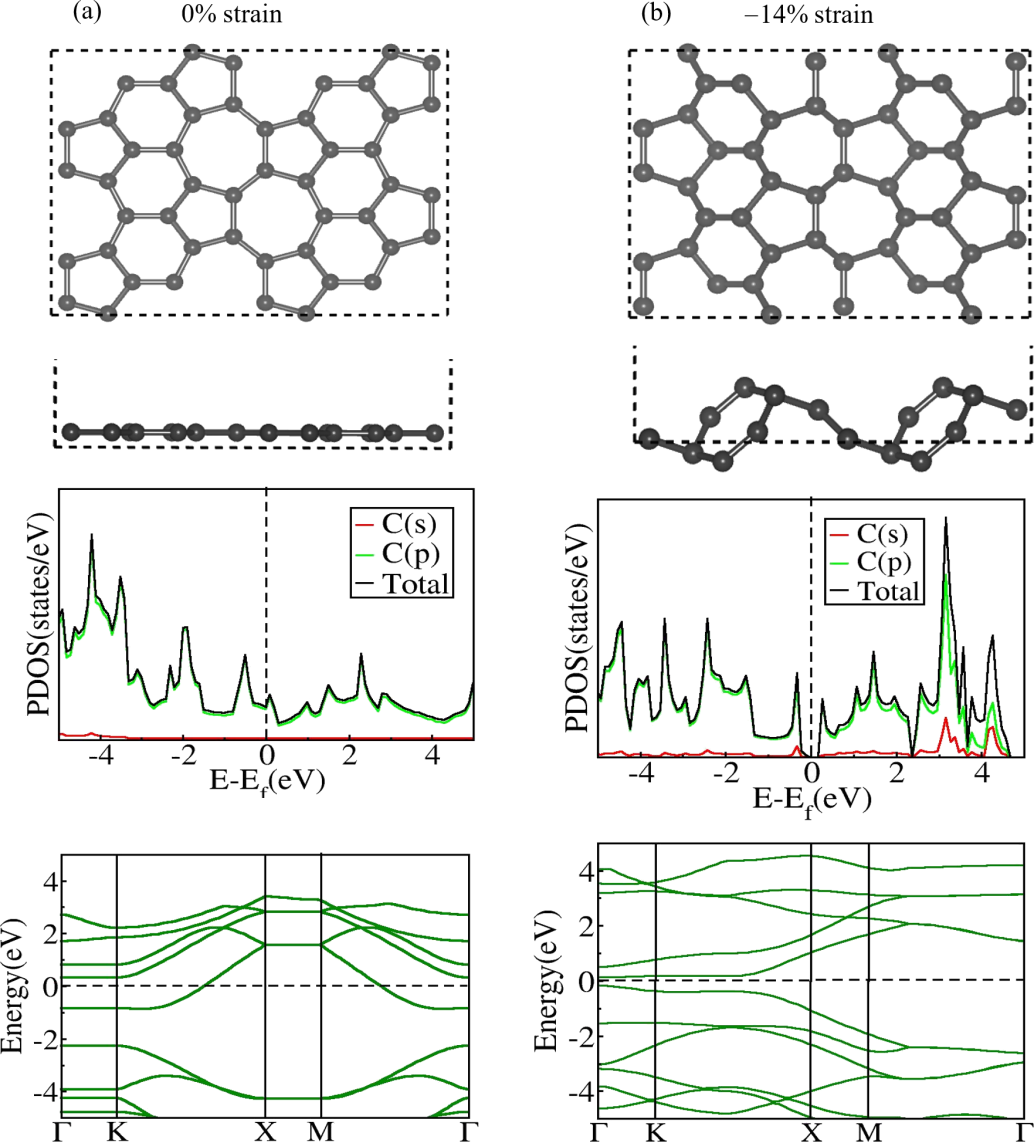
Relaxed 2 × 2 × 1 supercell’s top and side views, PDOS, and EBS of ψ-graphene (a) at 0% and (b) −14% strain. A direct-bandgap opening of 0.2 eV was found at −14% strain. We observe a buckling height of 2.13 Å at −14% applied strain.

We next investigated the impact of negative strain on the structural and electronic properties of ψ-graphene (Table 2). We show structure geometries, PDOS, and EBS of different negatively strained structures in Supporting Information File 1, Figures S1, S4, and S5, respectively. Our investigation reveals that, on progressively increasing the magnitude of applied negative strain in the lattice plane, ψ-graphene maintains its conductive nature until −13%; at −14%, a bandgap of ≈0.2 eV emerges between the valence and conduction bands as shown in Figure 2. Beyond −14% negative strain, a proportional increment in the bandgap is observed, reaching its maximum value of 0.7 eV at −16% strain. On surpassing −16% strain, the bandgap suddenly disappears at −17% strain (Figure S4, Supporting Information File 1). Beyond −17% strain, a significant distortion in the lattice structure of ψ-graphene was observed, which indicates a limit on the maximum strain that the ψ-graphene nanosheet can tolerate before structural breakdown. The EBS of ψ-graphene, plotted in Figure 2b at −14%, reveals a direct transition of electrons from the valence band to the conduction band, but beyond −14% strain, no direct transition is possible, indicating the indirect nature of the bandgap of ψ-graphene at strain levels of −15% and −6% (Table 2). The bond length *d*_1_, which was 1.51 Å in the absence of deformation, undergoes a considerable reduction under compression in the lattice plane. This bond length decreases until it reaches the lowest value of 1.27 Å at −10% strain level. The remaining C–C bond lengths fluctuate as the applied strain level increases or decreases.

It is known that the in-plane stiffness of ψ-graphene is higher than that of penta-graphene and is comparable to that of graphene [16]. Therefore, it can resist compressive strain (negative strain) in its lattice plane without experiencing much out-plane deformation or buckling. Here, we observe that ψ-graphene retains its flat structure up to −10% with negligible buckling, and only above this strain value buckling was observed in ψ-graphene sheets, as shown in Table 2 for −13% to −17% strain. Hence, within the negative strain range of −1% to −10%, ψ-graphene maintains its flat structural profile, but buckling appears beyond −10% strain. Notably, at the point of emergence of a bandgap (at −14%), the buckling becomes 2.13 Å (Table 2). Thus, in ψ-graphene, the bandgap remains zero with expansion (positive strain) along the lattice plane, while a negative strain of −14% generates a gap of 0.2 eV between its energy bands.

### Structural and electronic properties of ψ-graphone with strain

We tabulate the structural parameters, buckling heights, and electronic bandgap values of all strained structures in Table 3.

**Table 3:** Lattice parameters (*a*, *b*), C–C bond lengths (*d*_1_, *d*_C–C_), average C–C bond length (*d*_C–C_(avg)), buckling height (*h*), and bandgap energy (*E*_g_) of ψ-graphone on applying lattice strain.

Applied strain	Lattice parameters (Å)		*d* (Å)				*h* (Å)	*E*_g_ (eV)	Bandgap type
	
	*a*	*b*	*d*_1_	*d*_C–C_	*d*_C–C(avg)_	*d*_C–H_			

−16%	5.63	4.06	1.47	1.39–1.52	1.46	1.10–1.12	1.88	0.70	indirect
−15%	5.69	4.11	1.48	1.39–1.54	1.46	1.10–1.12	1.84	0.80	indirect
−13%	5.83	4.21	1.50	1.40–1.54	1.47	1.11–1.12	1.81	1.30	indirect
−10%	6.03	4.35	1.53	1.42–1.55	1.48	1.10–1.12	1.52	2.00	indirect
−7%	6.23	4.50	1.57	1.45–1.70	1.58	1.10–1.13	1.90	2.31	direct
−5%	6.36	4.59	1.57	1.44–1.57	1.51	1.10–1.13	1.52	2.31	direct
−3%	6.50	4.69	1.58	1.45–1.57	1.51	1.11–1.14	1.90	1.51	indirect
−2%	6.57	4.74	1.59	1.43–1.57	1.50	1.10–1.15	1.89	1.00	indirect
−1%	6.63	4.01	1.53	1.42–1.57	1.50	1.11–1.13	1.81	1.60	indirect
0%	6.70	4.83	1.61	1.47–1.57	1.52	1.11–1.14	1.79	0.00	–
1%	6.77	4.88	1.62	1.48–1.62	1.55	1.11–1.15	1.03	0.500	indirect
2%	6.83	4.93	1.64	1.43–1.59	1.51	1.11–1.15	0.98	0.300	indirect
3%	6.90	4.98	1.66	1.43–1.60	1.52	1.11–1.15	0.95	1.10	direct
5%	7.03	5.08	1.70	1.41–160	1.51	1.11–1.14	1.02	1.12	indirect
7%	7.17	5.17	1.77	1.37–1.62	1.50	1.10–1.12	1.00	2.10	direct
10%	7.37	5.46	2.17	1.42–1.73	1.58	1.10–1.12	1.02	0.300	direct
13%	7.57	5.46	2.05	1.47–1.78	1.62	1.11–1.12	1.03	2.00	indirect
15%	7.71	5.56	2.80	1.45–1.64	1.64	1.05–1.11	1.36	1.70	direct
16%	7.77	5.61	2.83	1.45–1.94	1.70	1.09–1.11	1.34	1.50	direct

#### Positive strain

**Figure 3:**
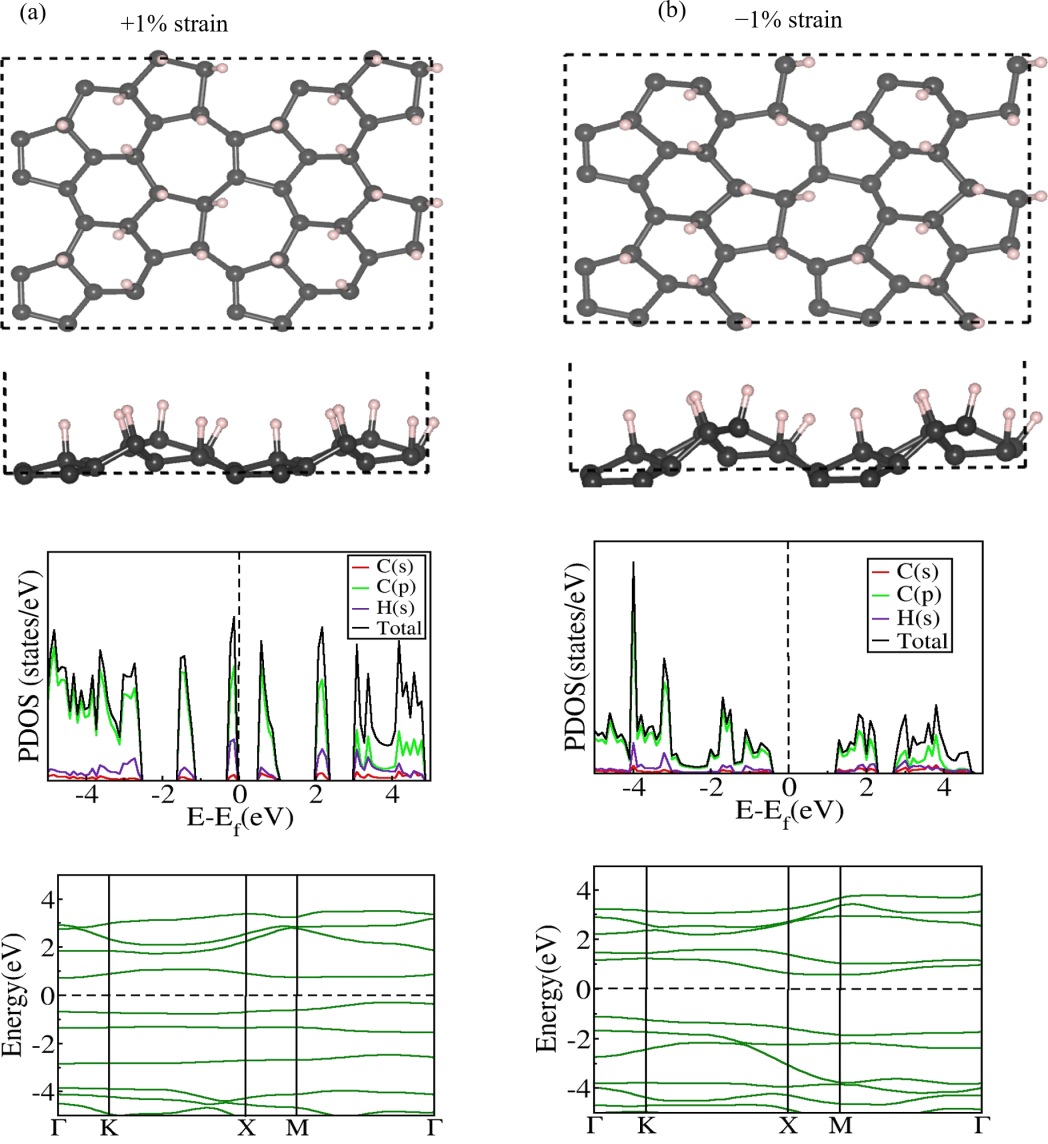
Relaxed 2 × 2 × 1 supercell’s top and side views, PDOS, and EBS of ψ-graphone (a) at +1% and (b) at −1% strain values. An opening of indirect bandgaps of 0.5 and 1.6 eV was found at +1% and −1% strain, respectively.

As mentioned earlier, like pristine ψ-graphene, partially hydrogenated ψ-graphene, ψ-graphone, is also a zero-bandgap material. Structures, PDOS, and EBS of all strained structures are given in Supporting Information File 1, Figures S6, S7, and S8, respectively. A bandgap opening of 0.5 eV was observed at the modest positive strain value of just +1% (Table 3, Figure 3), indicating that the electronic properties of ψ-graphone can be tuned with minimum structural deformation and low energy consumption. As can be seen from Table 3, the bandgap of ψ-graphone fluctuates with an increase in strain with a maximum bandgap of 2.10 eV at 7% strain. At this level, an elongation of 9.94% is found in *d*_1_ with a buckling height of 1.90 Å as shown in Figure S6 (Supporting Information File 1). Moreover, we also observed a transition from an indirect to a direct bandgap with a change in strain values applied to ψ-graphone sheets. Beyond the +10% strain level, suddenly, the separation between the bands increases to 2.00 eV, and on further increasing the magnitude of applied positive strain, it reduces to 1.50 eV at +16% strain value.

#### Negative strain

A remarkable observation is made regarding the electrical properties (Supporting Information File 1, Figures S9 and S10) when ψ-graphone undergoes negative mechanical strain ranging from −1% to −16% in its lattice plane. ψ-Graphone maintains its semiconducting nature within this negative strain range, and random separation is found between the valence and conduction bands of electrons, that is, within this negative strain range, the bandgap energies fluctuate for different strain values. The bandgap narrows at certain extents of deformation and widens at others. However, similar to positive strain, even a slight compression of −1%, which denotes a slight reduction in the lattice parameters, was found to be sufficient for a metal-to-semiconductor transition in ψ-graphone (Figure 3). These results indicate the extreme sensitivity of the ψ-graphone crystal structure to mechanical strain (tensile or compressive). As the magnitude of strain increases beyond −1%, the bandgap increases and reaches a stable value of 2.31 eV within a negative strain range of −5% to −7%. However, beyond the −7% strain level, a continuous reduction is observed in the bandgap, which continues up to −16% strain. At this level, the bandgap becomes 0.7 eV, signifying the smallest bandgap value and the limit of applying negative strain on ψ-graphone. Thus, initially, ψ-graphone transitions from a narrow bandgap, proof of its semiconducting behavior, to a wide bandgap. However, on further increasing the negative strain, it exhibits a reversal in its bandgap from a wider gap to a narrower one. Another intriguing observation shown in Table 3 is the nature of the bandgap at various negative strain values. Only in the specific span of −5% to −7%, where the bandgap becomes stable, a direct transition of electrons from the valence to the conduction band is allowed. Outside this strain range, ψ-graphone has an indirect bandgap. Furthermore, the buckling height of ψ-graphone increased from initially 1.79 to 1.90 Å at strain levels of −3% and −7%, signifying its lowest mechanical stability at these strain levels. Although the presence of negative strain affects the lengths of all C–C bonds of ψ-graphone, the bond length *d*_1_ at the junction of the *s*-indacene molecule reaches its minimum value of 1.47 Å at −16% strain along the lattice plane, which indicates that only 8.69% contraction is possible in this specific bond length of ψ-graphone. The bond length between C atoms and attached H atoms typically ranges from 1.05 to 1.15 Å.

### Structural and electronic properties of ψ-graphane with strain

We tabulate the structural parameters, buckling height, and electronic bandgap values of all strained structures in Table 4.

**Table 4:** Lattice parameters (*a*, *b*), C–C bond lengths (*d*_1_, *d*_C–C_), average C–C bond length (*d*_C–C_(avg)), buckling height (*h*), and bandgap energy (*E*_g_) of ψ-graphane on applying lattice strain.

Applied strain	Lattice parameters (Å)		*d* (Å)				*h* (Å)	*E*_g_ (eV)	Bandgap type
	
	*a*	*b*	*d*_1_	*d*_C–C_	*d*_C–C(avg)_	*d*_C–H_			

−17%	5.56	4.01	1.43	1.33–1.54	1.43	1.10–1.11	2.02	1.40	direct
−16%	5.63	4.06	1.43	1.35–1.53	1.44	1.10–1.11	1.96	1.50	direct
−15%	5.69	4.11	1.42	1.35–1.46	1.41	1.10–1.11	1.98	1.80	direct
−13%	5.83	4.21	1.43	1.36–1.51	1.44	1.10–1.11	1.92	1.90	direct
−10%	6.03	4.35	1.45	1.38–1.50	1.44	1.10–1.11	1.70	2.21	direct
−7%	6.23	4.50	1.48	1.41–1.49	1.45	1.10–1.11	1.42	2.70	direct
−5%	6.36	4.59	1.50	1.44–1.50	1.47	1.10–1.11	1.01	3.00	direct
−3%	6.50	4.69	1.53	1.45–1.51	1.48	1.10–1.11	0.85	3.30	direct
−2%	6.57	4.74	1.54	1.47–1.51	1.49	1.10–1.11	0.97	3.41	direct
−1%	6.63	4.01	1.55	1.49–1.52	1.51	1.10–1.11	0.91	3.61	direct
0%	6.70	4.83	1.56	1.49–1.55	1.52	1.10–1.11	0.85	3.78	direct
1%	6.77	4.88	1.58	1.50–1.54	1.52	1.10–1.11	0.85	3.80	direct
2%	6.83	4.93	1.59	1.51–1.56	1.54	1.10–1.11	0.81	3.90	direct
3%	6.90	4.98	1.61	1.53–1.58	1.56	1.10–1.11	0.78	4.00	direct
5%	7.03	5.08	1.64	1.54–1.63	1.59	1.10–1.11	0.74	4.11	direct
7%	7.17	5.17	1.67	1.56–1.63	1.60	1.10–1.11	0.70	4.31	direct
10%	7.37	5.46	1.76	1.61–1.68	1.65	1.10–1.11	0.65	4.50	direct
13%	7.57	5.46	1.75	1.61–1.72	1.66	1.10–1.11	0.63	4.70	direct
15%	7.71	5.56	1.78	1.62–1.75	1.68	1.10–1.11	0.62	4.70	direct
16%	7.77	5.61	1.79	1.64–1.76	1.70	1.10–1.11	0.61	4.81	direct
17%	7.84	5.66	1.80	1.64–1.78	1.71	1.10–1.11	0.61	4.81	direct

#### Positive strain

**Figure 4:**
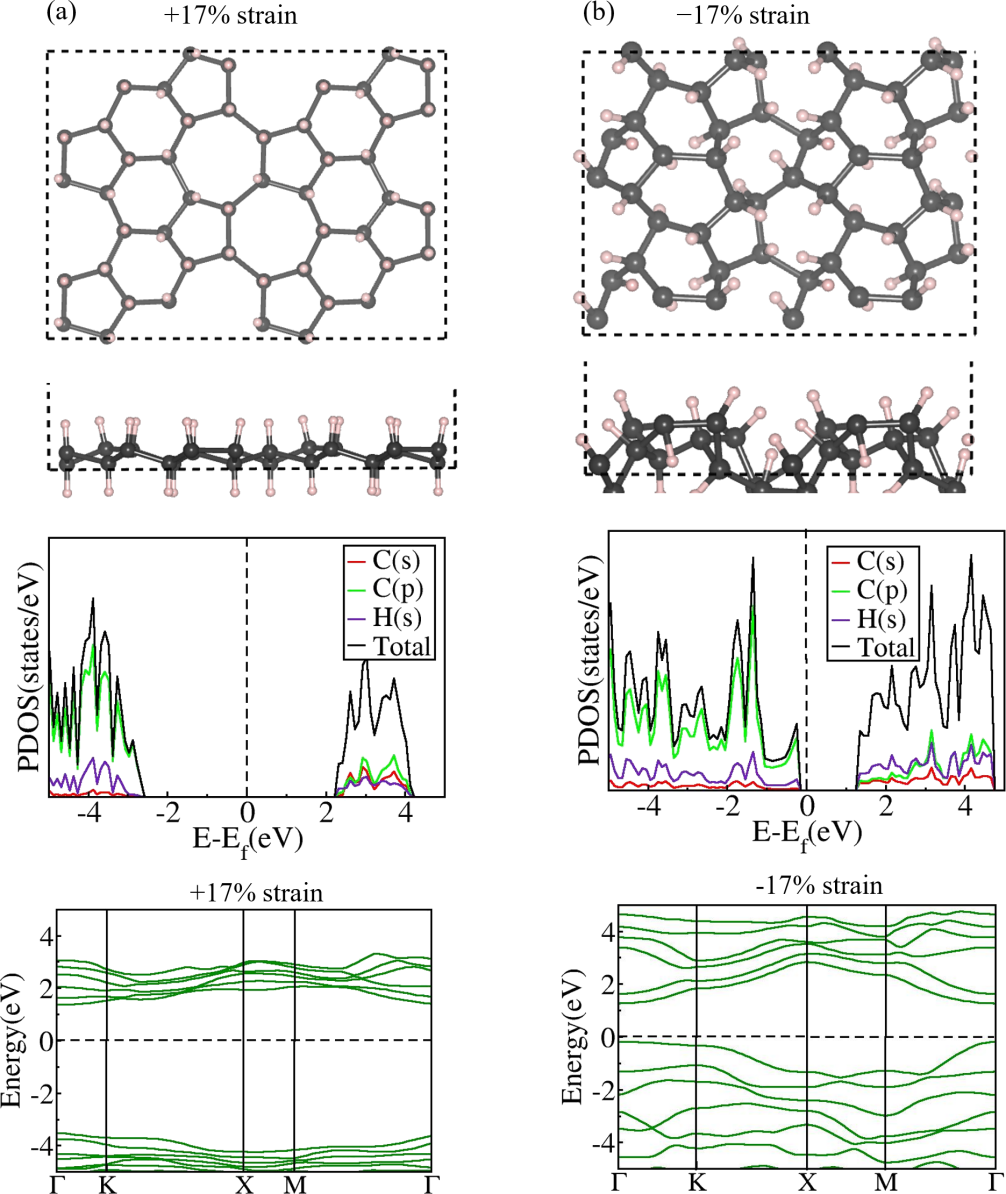
Relaxed 2 × 2 × 1 supercell’s top and side views, PDOS, and EBS of ψ-graphane (a) at −17% and (b) +17% strain values. The bandgap of ψ-graphane becomes maximum at +17% strain (4.81 eV) and minimum at −17% (1.40 eV) strain values. The type of the bandgap at both strain levels was observed to be direct.

Next, we applied positive mechanical strain to fully hydrogenated ψ-graphene, that is, ψ-graphane (Supporting Information File 1, Figure S11). As mentioned earlier, a bandgap of 3.78 eV is observed in the pristine ψ-graphane sheet. When ψ-graphane undergoes positive strain, the C–C bonds expand freely, leading to an increment in *d*_1_ from 1.56 Å to 1.80 Å at a strain level of +17% as shown in Table 4, where we tabulate structural parameters and bandgap of all strained structures. Unlike ψ-graphone, the mechanical strain does not affect the C–H bond length, which remains fixed at 1.10–1.11 Å, indicating the formation of strong C–H bonds in ψ-graphane compared to ψ-graphone. We note a linear bandgap increase in ψ-graphane from 3.78 to 4.81 eV when the strain is increased from 0 to 17% (Figures S12 and S13, Supporting Information File 1). Furthermore, ψ-graphane exhibits a direct bandgap at all applied strain values, and electrons cannot move from the valence to the conduction band even when strain is applied. This direct bandgap allows the most efficient transport of charge carriers and easy recombination of electrons and holes, indicating its suitability in quantum computing, which requires semiconductors with direct bandgaps. ψ-Graphane can endure up to +17% mechanical strain (Figure 4) suggesting that the bandgap of ψ-graphane can be increased up to 27.25% by positive mechanical strain. Unlike ψ-graphone, which shows fluctuations in buckling height, the buckling height of fully hydrogenated ψ-graphene consistently decreases on increasing the applied positive strain. It reduces to 0.61 Å at both +16% and +17% strain levels.

#### Negative strain

The cell parameters of ψ-graphane can be compressed by imposing a negative mechanical strain up to −17% from *a* = 6.70 Å and *b* = 4.83 Å to *a* = 5.56 Å and *b* = 4.01 Å (Table 4, Supporting Information File 1, Figure S11). ψ-graphane can tolerate a mechanical strain range from −17% to +17% before experiencing structural distortion. Introducing negative mechanical strain to ψ-graphane, akin to the effect of positive mechanical strain, does not result in the disappearance of the electronic bandgap at any strain level. Additionally, despite the presence of negative mechanical strain, the type of bandgap of ψ-graphane remains direct, similar to the positive strain effect in ψ-graphane; however, a continuous reduction in bandgap was observed with an increase in negative strain values. At −17% strain level, the bandgap (Figure 4) and *d*_1_ converge to 1.40 eV and 1.43 Å, respectively (Table 4), while the buckling height increases to 2.02 Å. Neither exposing ψ-graphane to negative strain yielded any discernible alteration in the C–H bond lengths, nor did the application of positive strain. Throughout the range from −17% to +17%, ψ-graphane exhibits the behavior of a wide-bandgap semiconductor (Figures S14 and S15, Supporting Information File 1). The carbon p orbitals provide the major contribution to the valence band. In contrast, in the conduction band, the s orbitals of carbon and hydrogen atoms and the p orbitals of carbon atoms contribute equally.

**Figure 5:**
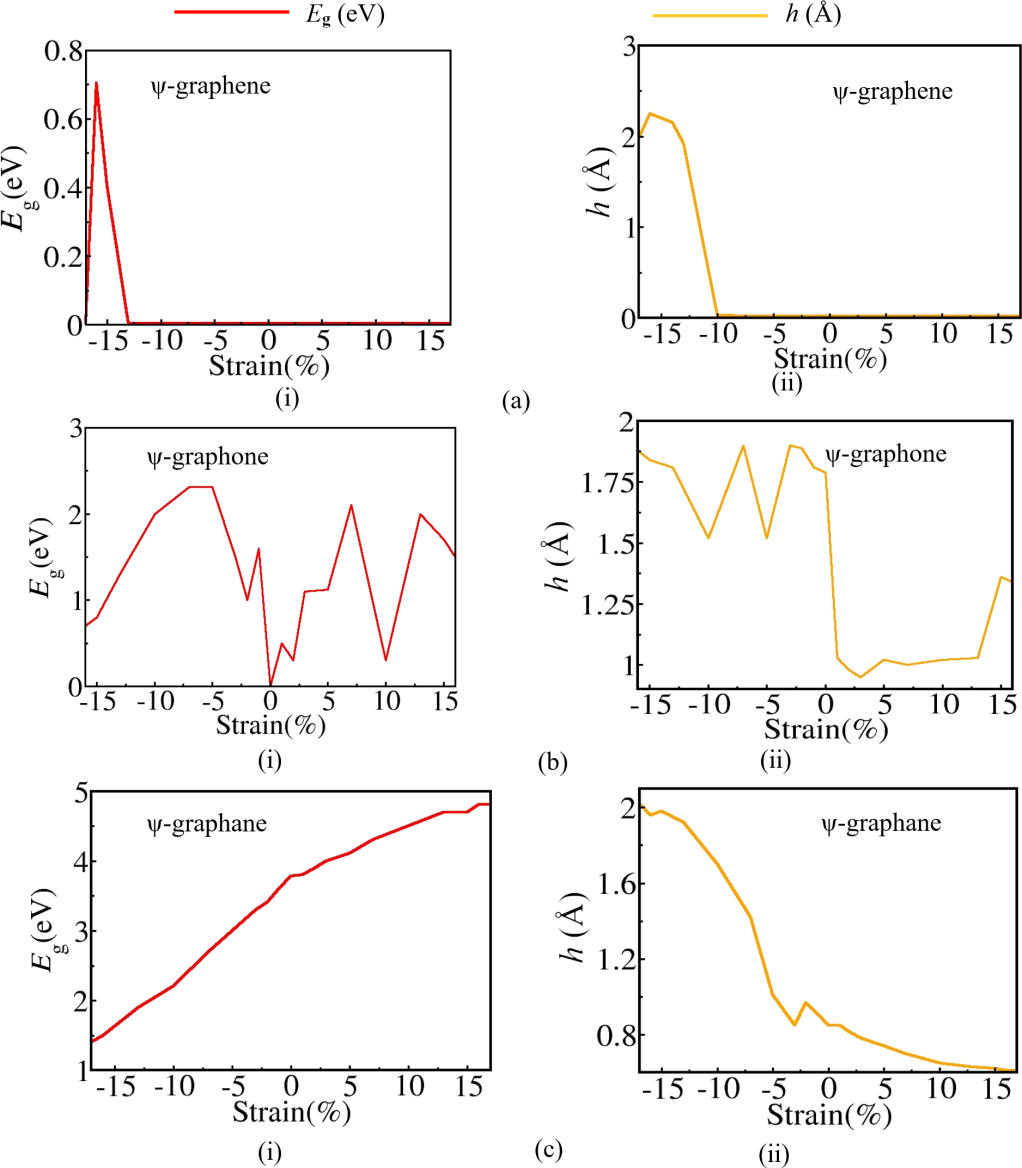
Variation of (i) bandgap energy *E*_g_ and (ii) buckling height *h* of (a) ψ-graphene, (b) ψ-graphone, and (c) ψ-graphane under applied biaxial mechanical strain. The bandgap *E*_g_ and buckling height *h* of ψ-graphane vary in an almost linear fashion.

We have plotted the variation in bandgap and buckling height in ψ-graphene, ψ-graphone, and ψ-graphane 2D sheets in Figure 5. It can be seen easily that in ψ-graphene, the application of negative strain opens up a bandgap of 0.2 eV at −14%, which reaches a maximum value of 0.7 eV at −16% strain with a buckling height of 2.3 Å. Here, positive strain fails to open the bandgap. In ψ-graphone, a fluctuation in the bandgap is observed with a bandgap opening at 1% values of positive and negative strain. In ψ-graphane, the bandgap increases linearly with applied positive strain from its original value of 3.78 to 4.81 eV at 17%, while a linear decrease in bandgap is observed with negative strain.

## Conclusion

In this study, we explored the structural and electronic properties of ψ-graphene and its partially and fully hydrogenated forms ψ-graphone and ψ-graphane, respectively, upon introducing mechanical strain. We observed that strain engineering affects the bandgap of pristine and hydrogenated ψ-graphene 2D nanosheets. In summary, positive strain along the lattice plane of ψ-graphene cannot create a gap between its energy bands. However, at a negative mechanical strain of around −14%, a bandgap of 0.2 eV becomes apparent in the band structure of ψ-graphene, changing it from zero-bandgap to a narrow-bandgap semiconductor. These results signify ψ-graphene’s low bandgap sensitivity to mechanical strain. Enhanced sensitivity to mechanical strain was observed in ψ-graphone, a zero-bandgap material. We found that the bandgap of ψ-graphone can be opened even under a slight strain of −1% or +1%, highlighting the remarkable sensitivity of ψ-graphone towards mechanical deformation. In contrast, ψ-graphane is a direct-bandgap material that remains unchanged under mechanical strain. This outcome offers various critical applications of ψ-graphane in photodetectors, solar cells, LEDs, pressure and strain sensors, energy storage, and quantum computing. The mechanical strain tolerance of pristine and fully hydrogenated ψ-graphene is observed to be −17% to +17%, whereas for ψ-graphone, it lies within the strain span of −16% to +16%. The remarkable strain tolerance of these materials makes them promising candidates for flexible displays and other electronic devices.

## Computational Methodology

The computational parameters used in our calculations are based on the density functional theory (DFT) as implemented in Quantum Espresso [46]. Our computations employed the generalized gradient approximation (GGA) and Perdew–Burke–Ernzerhof exchange–correlation functionals [47]. The crystal structure of pristine and hydrogenated ψ-graphene has the space group *P*2*mg* [39]. The unit cell of ψ-graphene contains 12 carbon atoms. In comparison, the unit cells of ψ-graphone and ψ-graphane consist of 12 carbons and six hydrogens and 12 carbons and 12 hydrogen atoms, respectively (Figure 1) [38–39]. For the sampling of the Brillouin zone, we used a well converged 8 × 8 × 1 k-point mesh, and the electronic wave functions were expanded within a basis set of plane waves with a 600 eV cutoff energy. The unwanted interactions between the periodic images of 2D sheets have been mitigated by incorporating a generous vacuum space of 13 Å into our simulation cell. The convergence in self-consistency was achieved at a stringent threshold energy value of 10^−5^ eV, and forces acting on atoms were converged to 0.01 eV.

## Supporting Information

We have given relaxed structures, PDOS, and EBS plots of ψ-graphene, ψ-graphone, and ψ-graphane in the Supporting Information. We have also given variations of average bond lengths and *d*_1_ bond lengths with applied uniform biaxial mechanical strain in these three materials.

File 1 Additional figures.

## Data Availability

All data that supports the findings of this study is available in the published article and/or the supporting information to this article.
