# Supplementary material for: Strain-induced bandgap engineering in 2D ψ-graphene materials: a first-principles study
Source: Beilstein J Nanotechnol. 2024 Nov 20;15:1440–52. doi: 10.3762/bjnano.15.116 (PMC11590022; doi:10.3762/bjnano.15.116)
Supplement: File 1 — Additional figures. [file Beilstein_J_Nanotechnol-15-1440-s001.pdf]

BEILSTEIN JOURNAL OF NANOTECHNOLOGY

Supporting Information

for

Strain-induced bandgap engineering in 2D ψ-graphene materials: a first-principles study

Kamal Kumar, Nora H. de Leeuw, Jost Adam and Abhishek Kumar Mishra

Beilstein J. Nanotechnol. **2024,** *15,* 1440–1452. doi:10.3762/bjnano.15.116

Additional figures

License and Terms: This is a supporting information file under the terms of the Creative Commons Attribution License (https://creativecommons.org/licenses/by/4.0). Please note that the reuse, redistribution and reproduction in particular requires that the author(s) and source are credited and that individual graphics may be subject to special legal provisions.
The license is subject to the *Beilstein Journal of Nanotechnology* terms and conditions: (https://www.beilstein-journals.org/bjnano/terms)

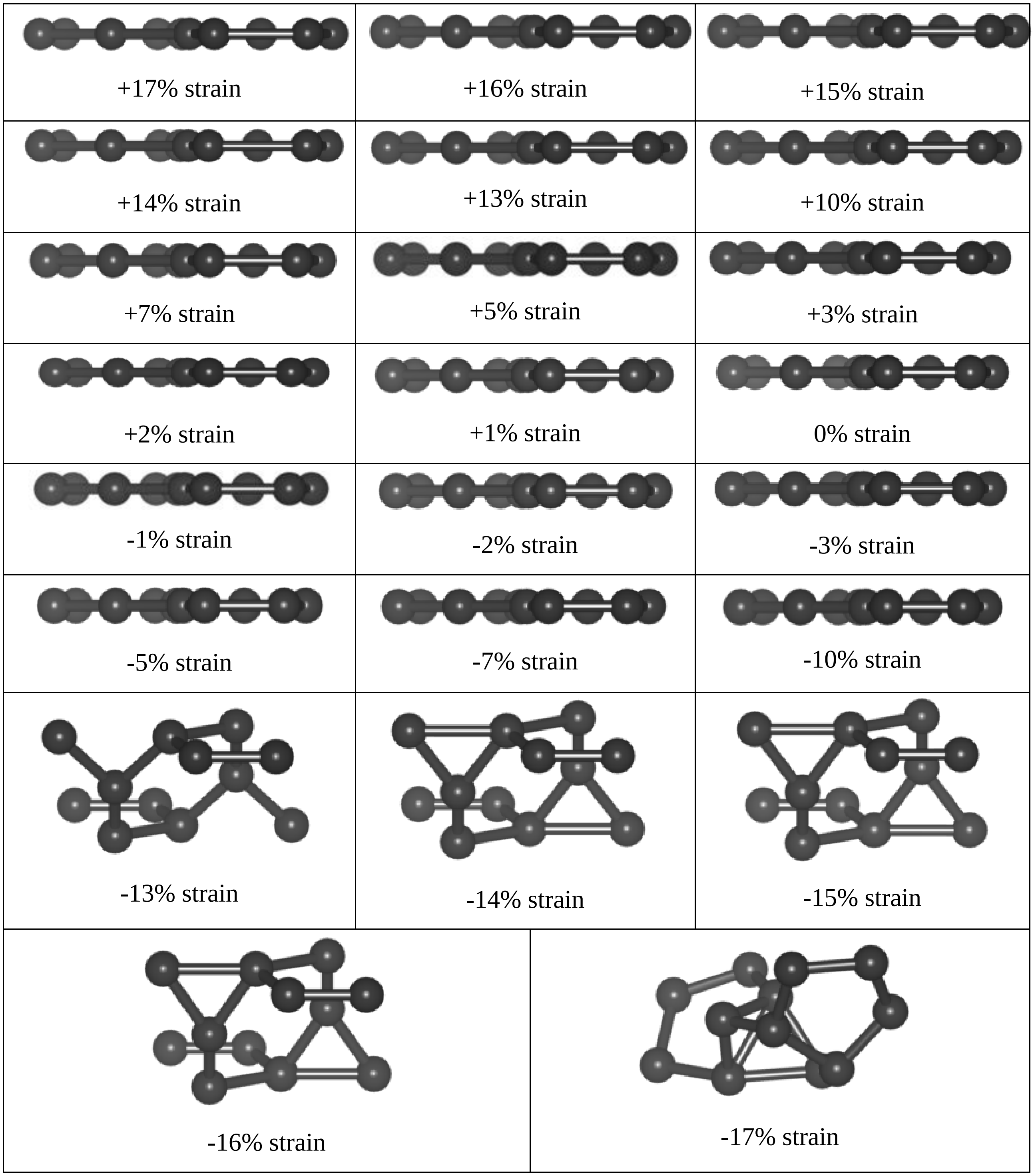

Figure S1: Side view of the relaxed unit cell of ψ-graphene at different strain values.

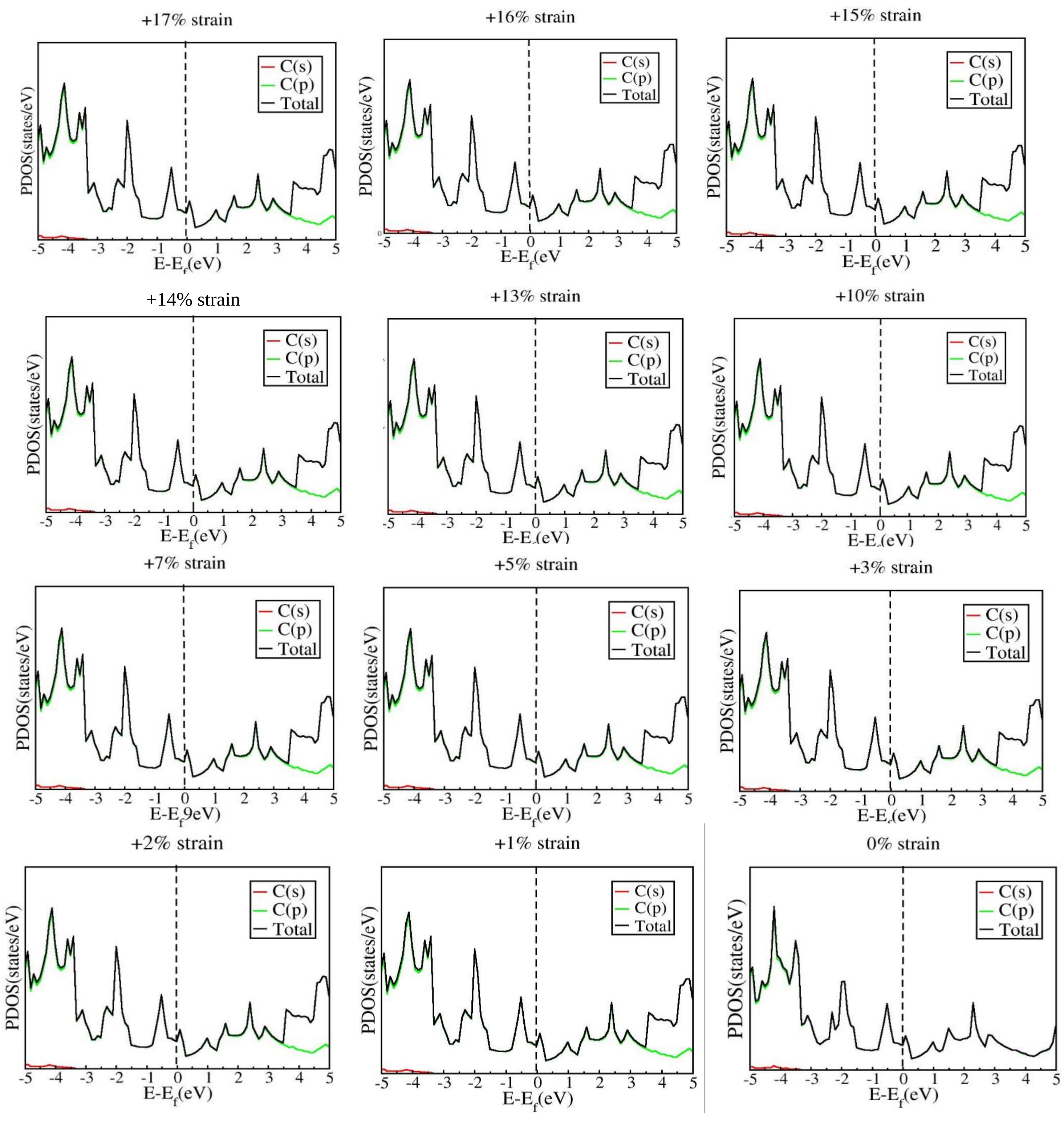

Figure S2: PDOS of ψ-graphene at different values of positive strain.

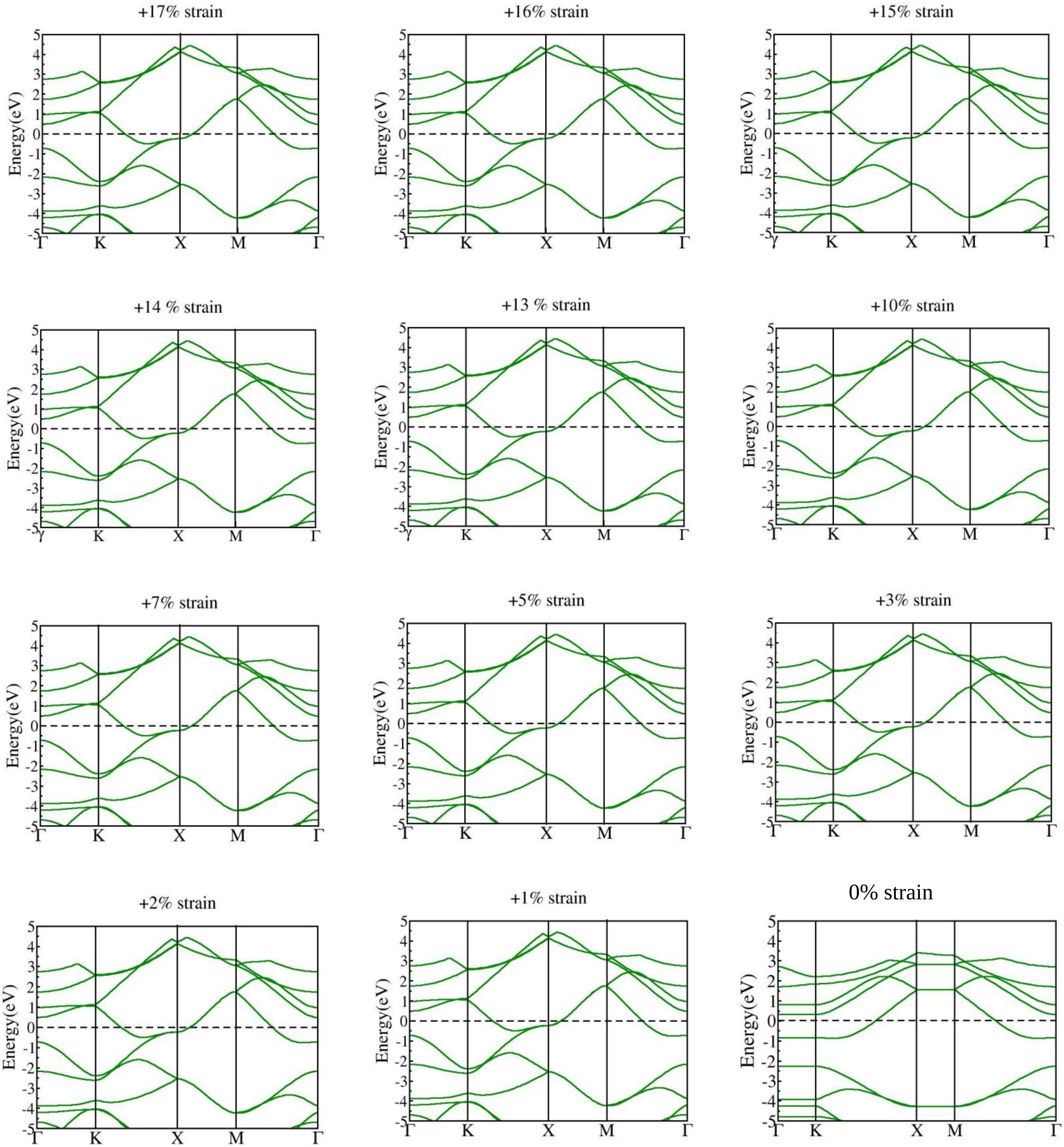

Figure S3: Electronic band structure of ψ-graphene at different values of positive strain.

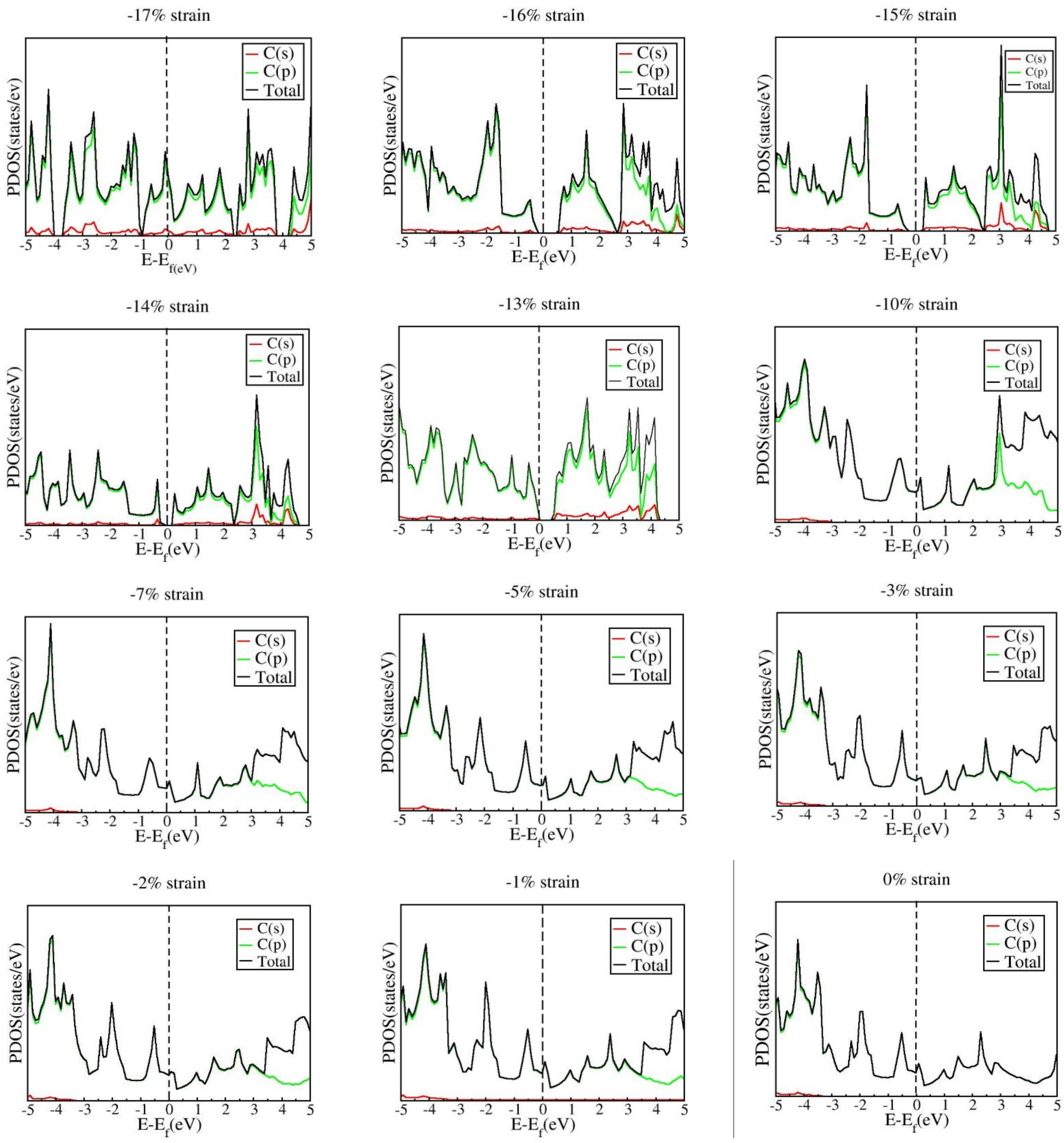

Figure S4: PDOS of ψ-graphene at different values of negative strain.

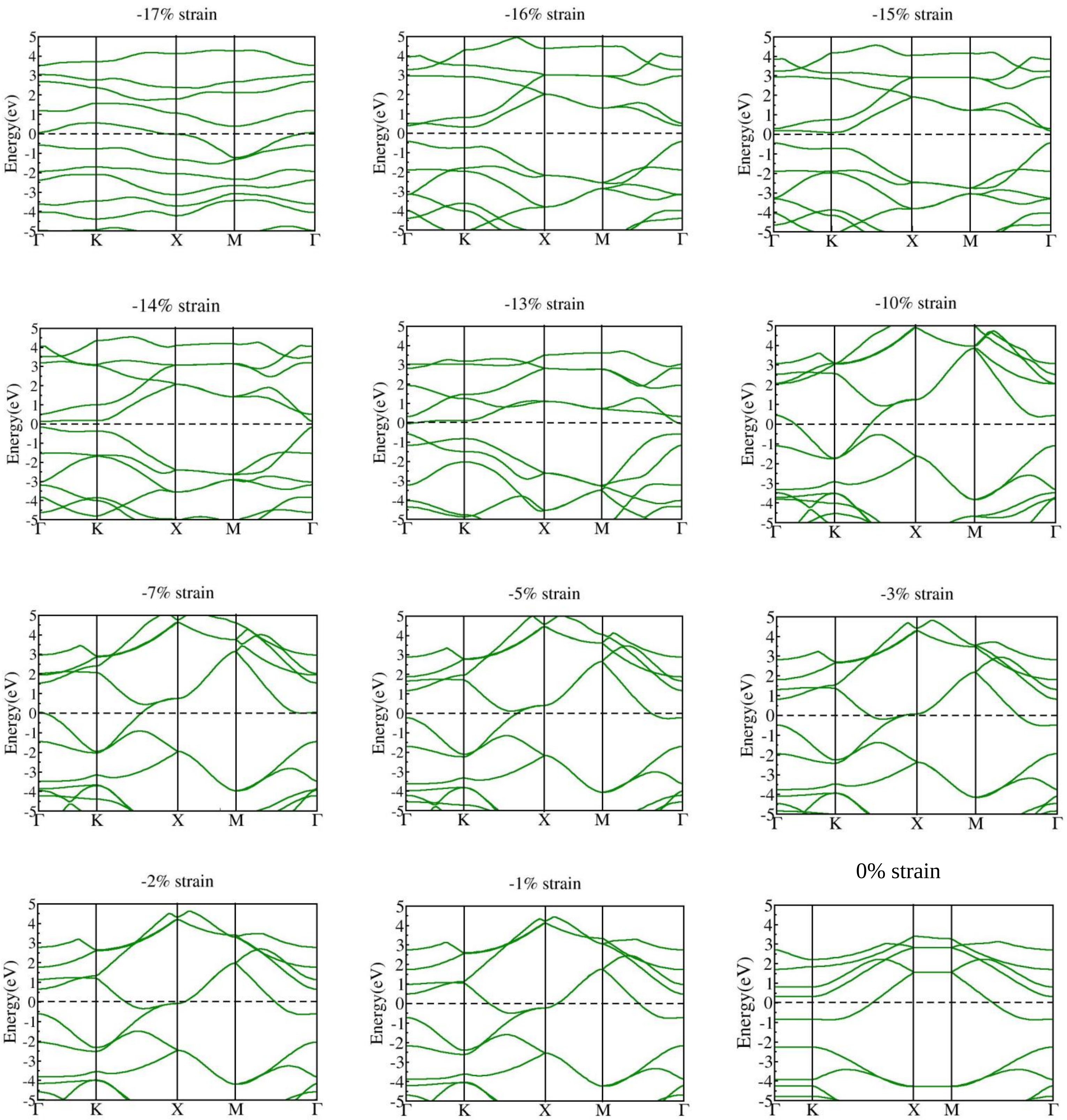

Figure S5: Electronic band structure of ψ-graphene at different values of negative strain.

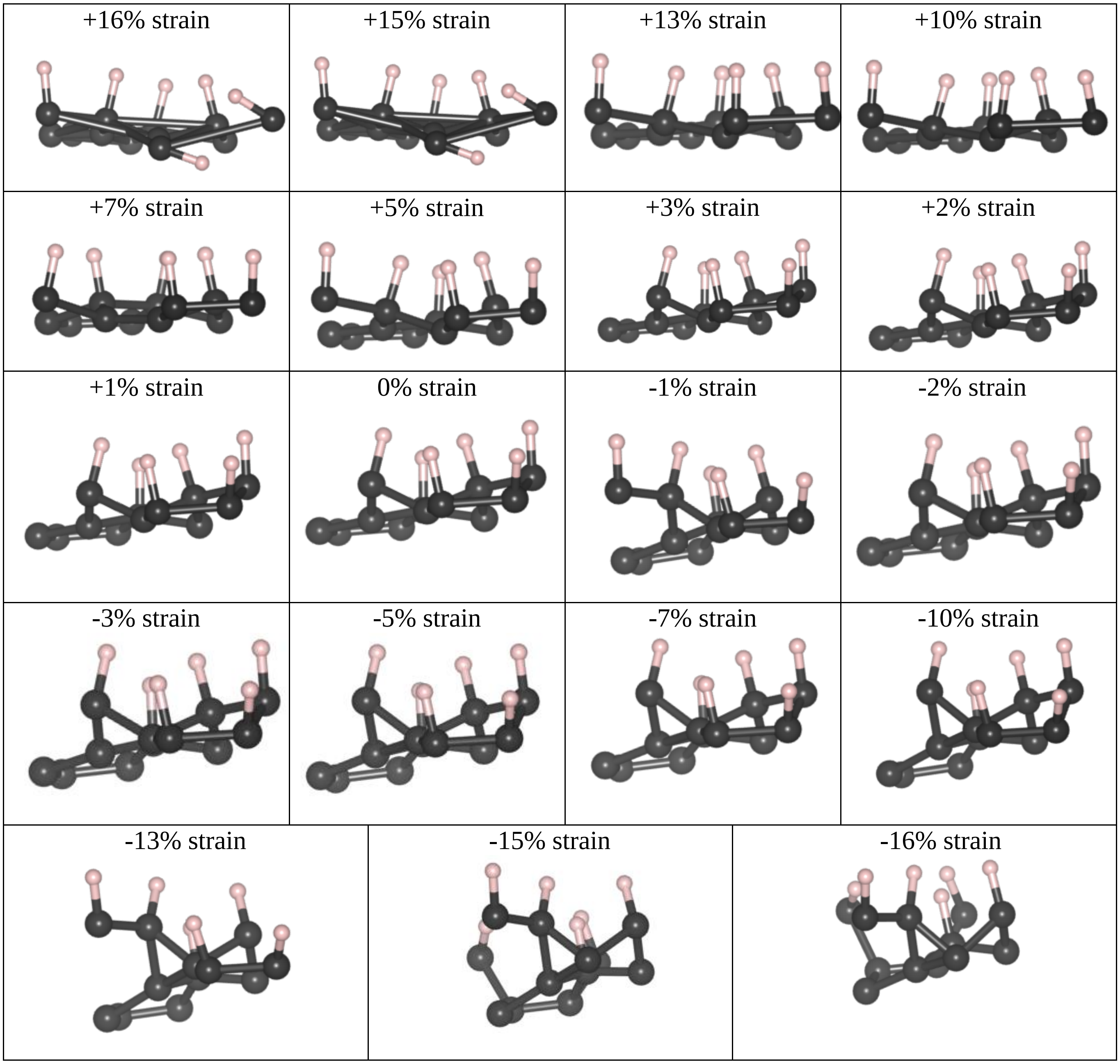

Figure S6: Side view of the relaxed unit cell of ψ-graphone at different strain values.

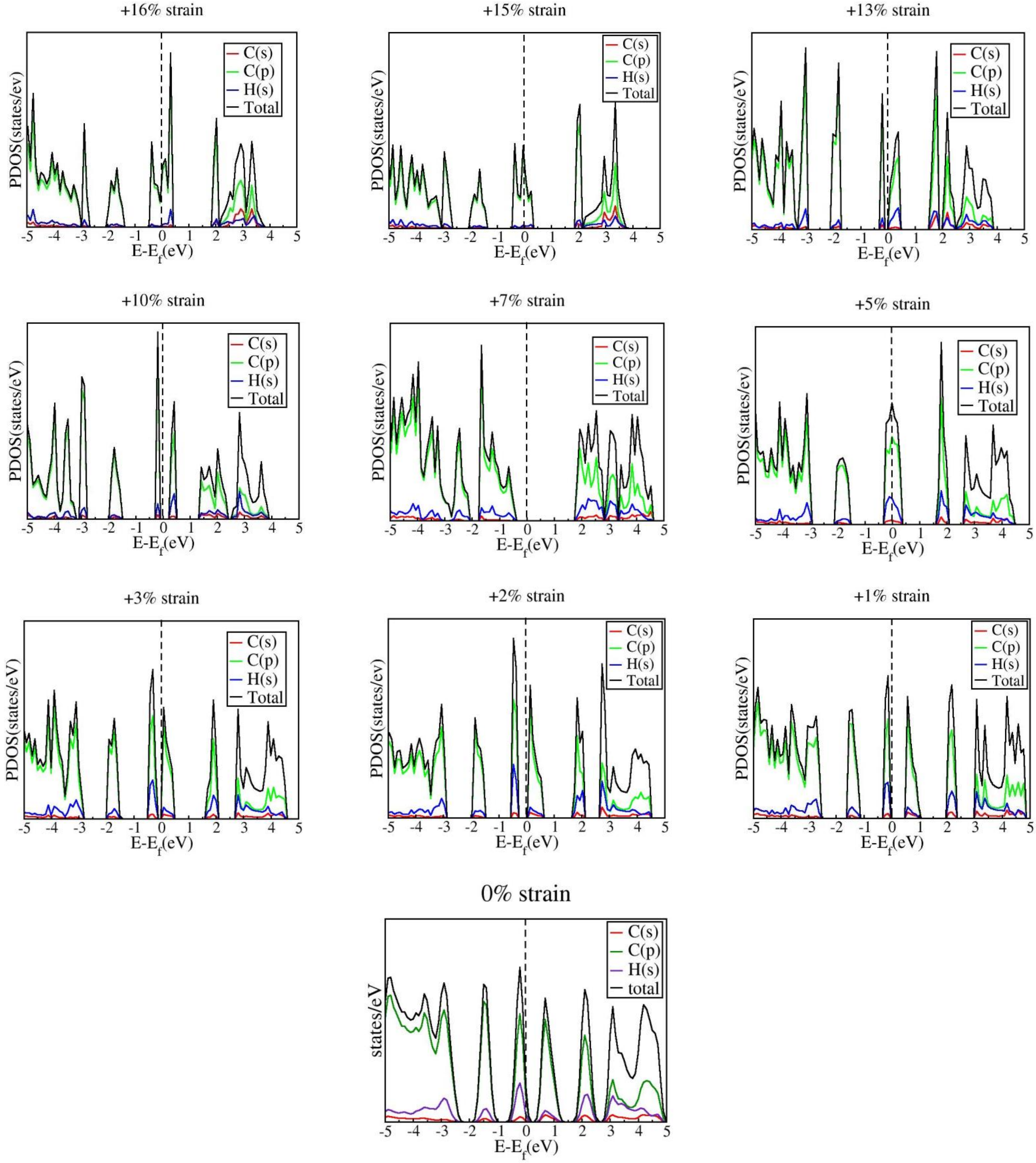

Figure S7: PDOS of ψ-graphone at different values of positive strain.

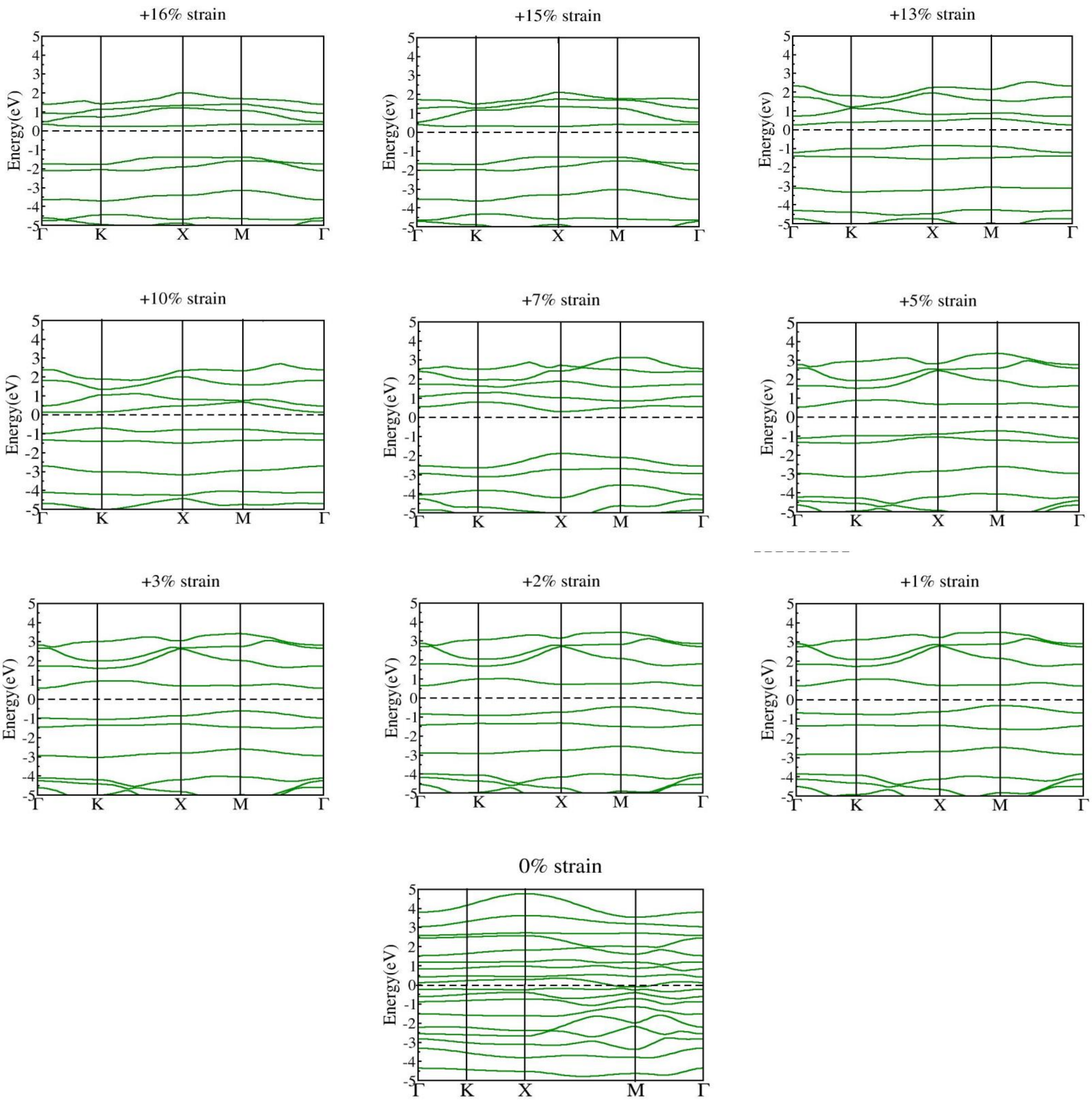

Figure S8: Electronic band structure of ψ-graphone at different values of positive strain.

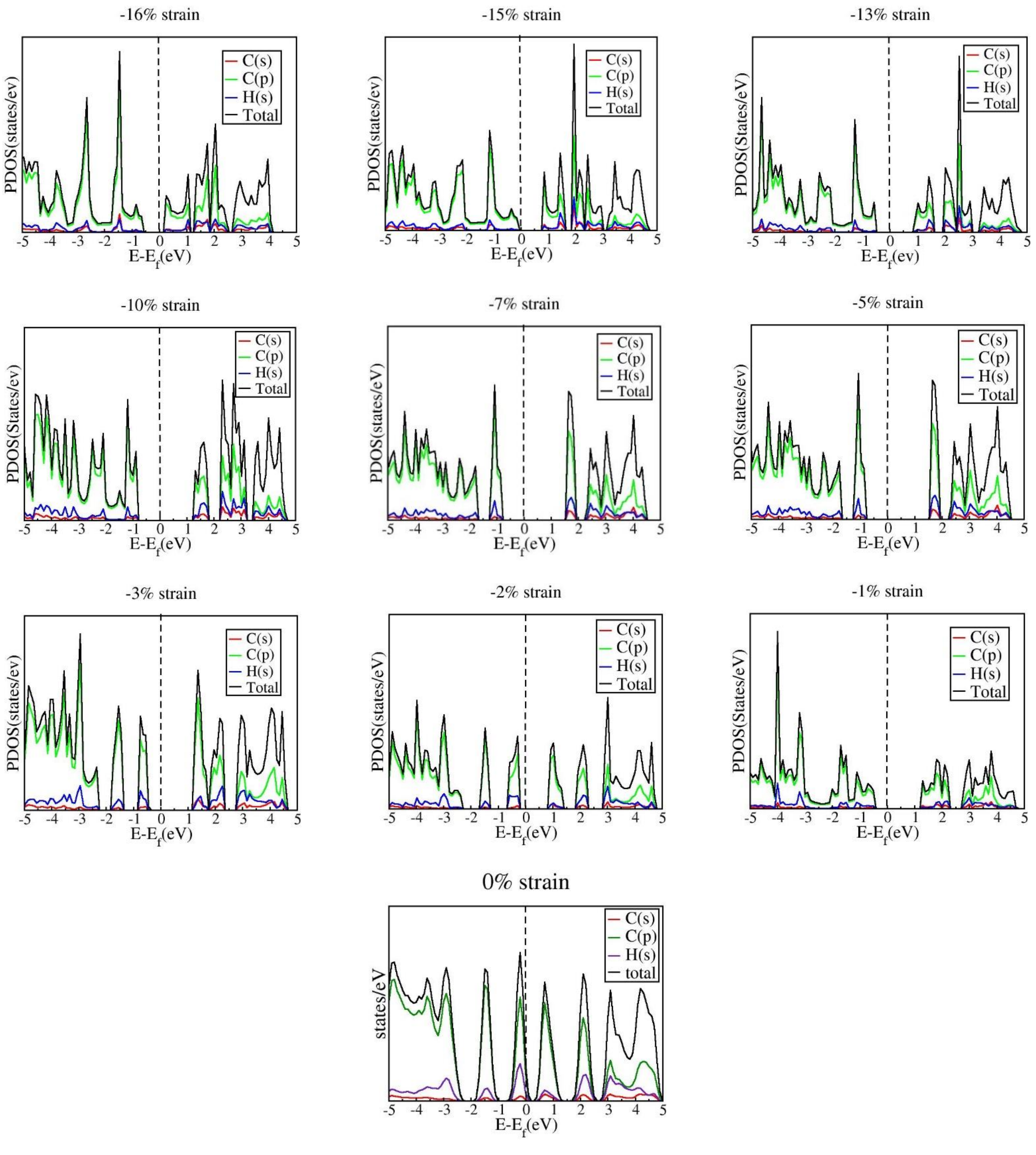

Figure S9: PDOS of ψ-graphone at different values of negative strain.

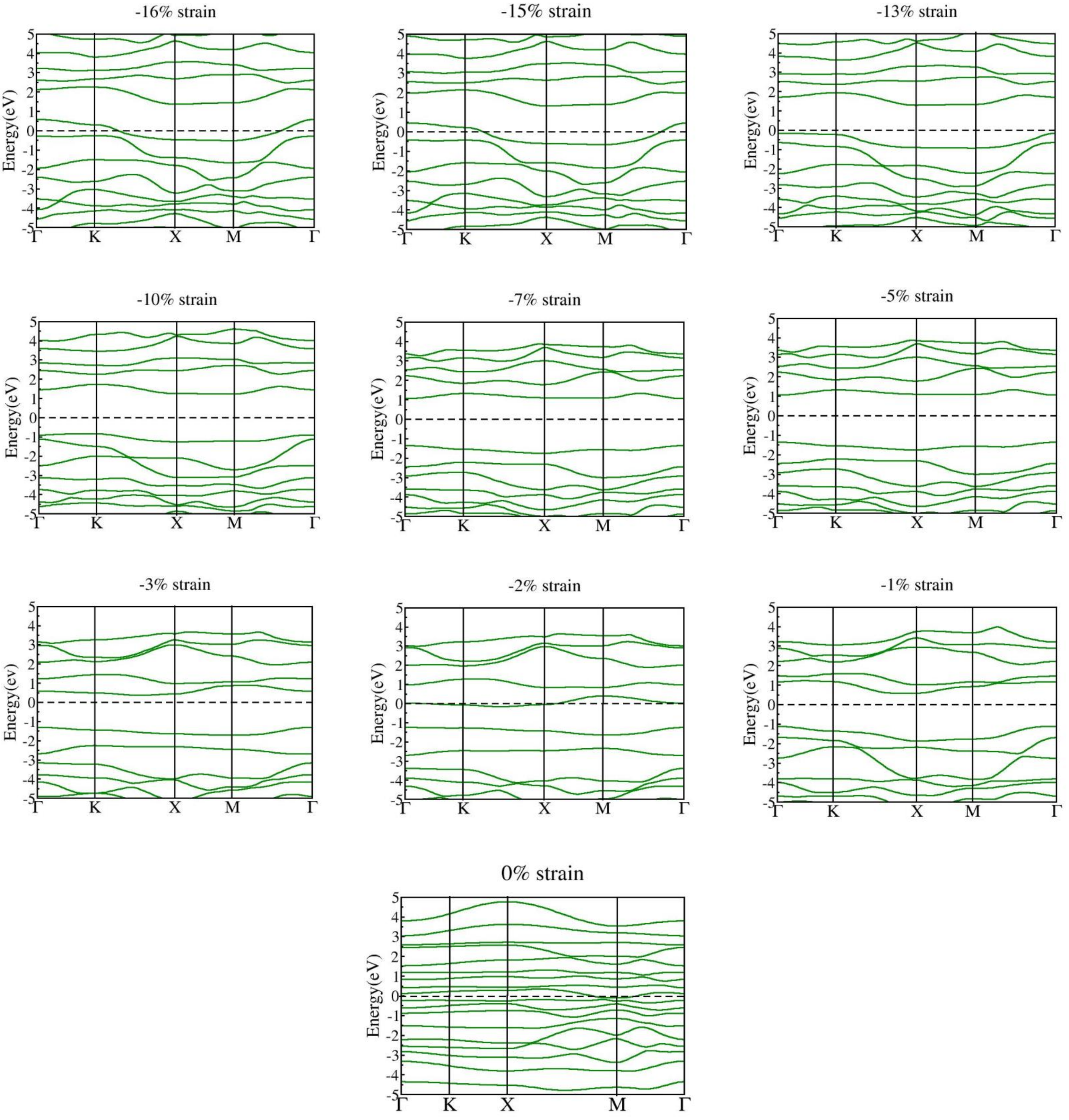

Figure S10: Electronic band structure of ψ-graphone at different values of negative strain.

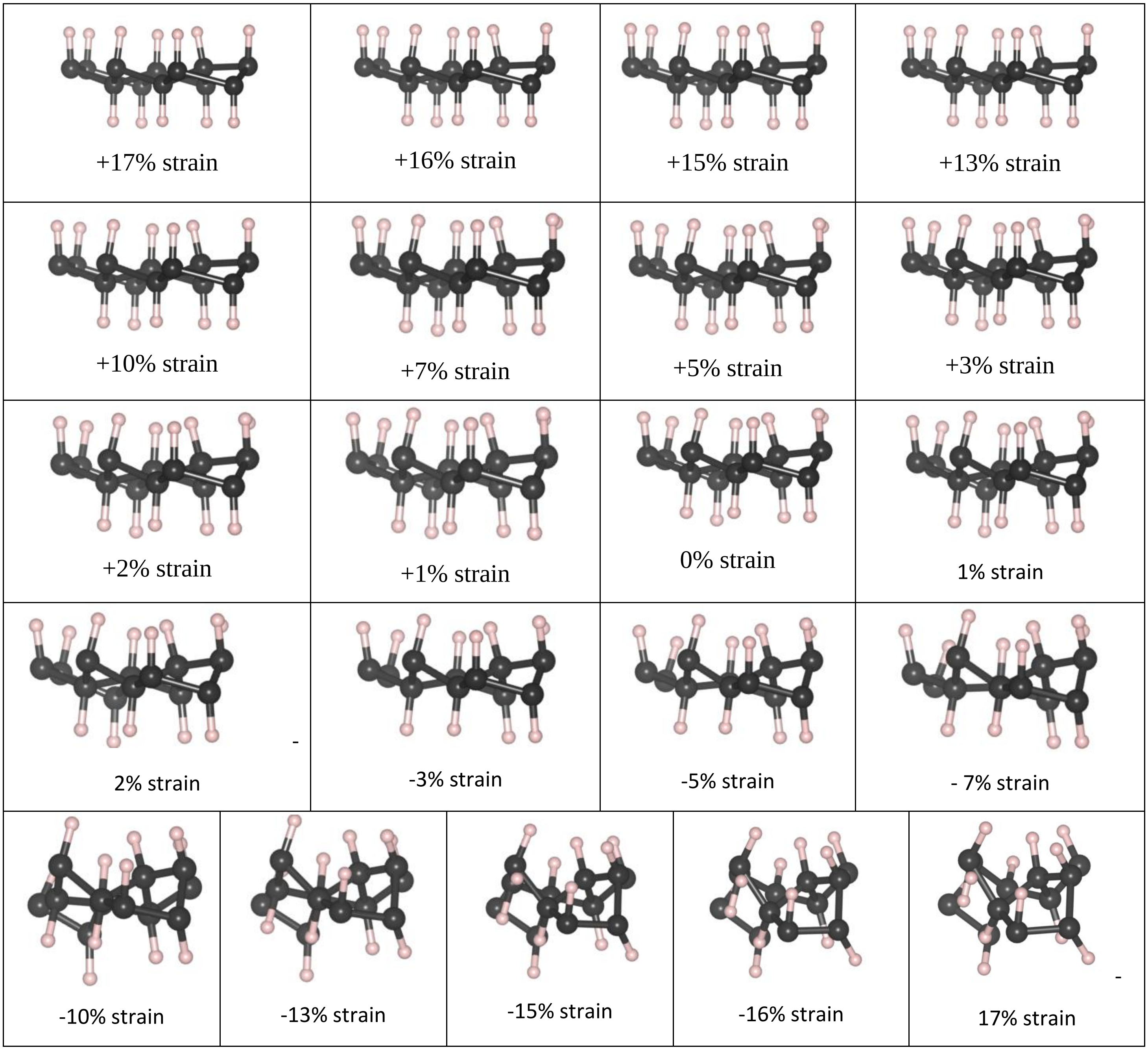

Figure 11: Side view of the relaxed unit cell of ψ-graphane at different strain levels.

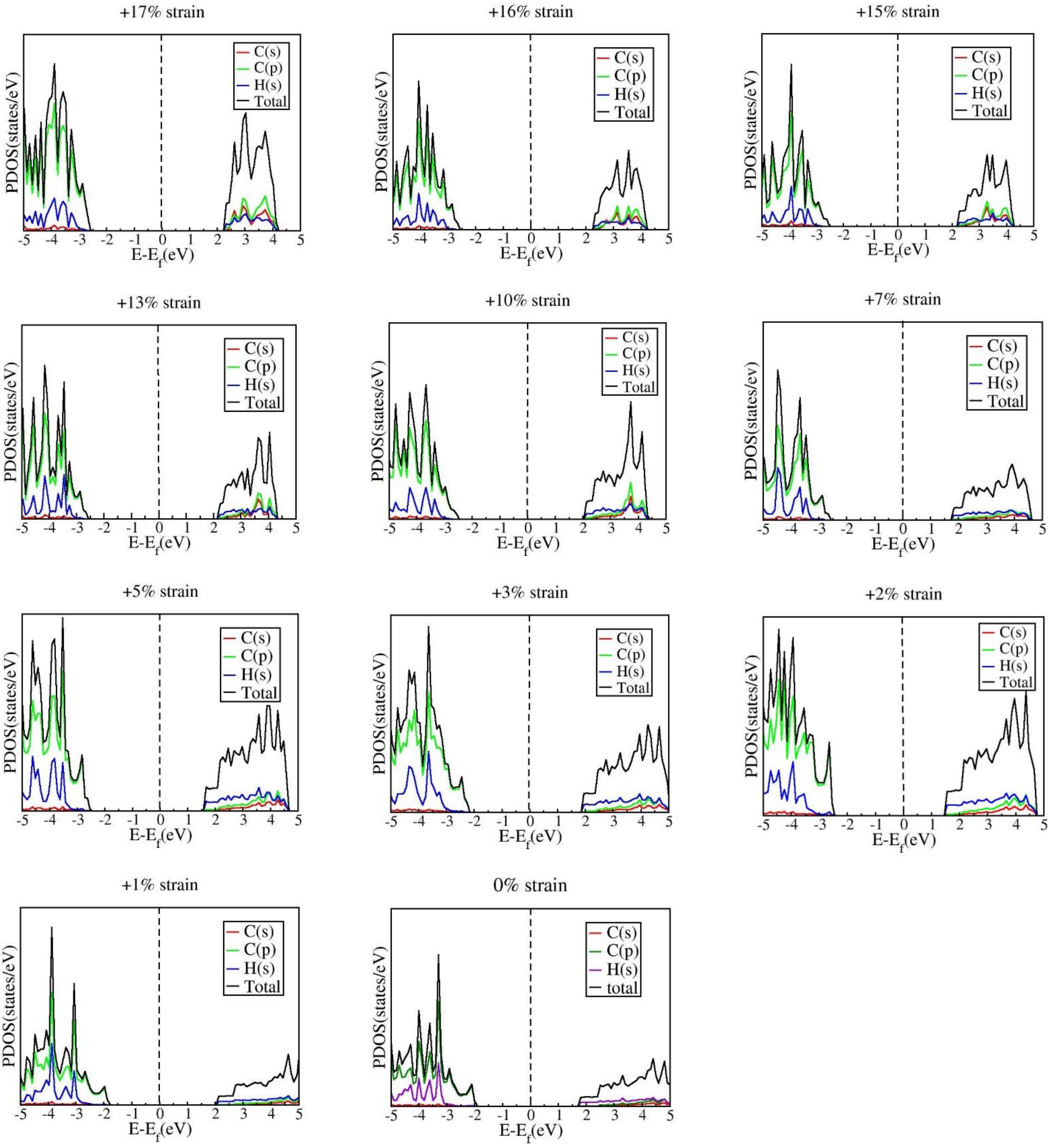

Figure S12: PDOS of ψ-graphane at different values of positive strain.

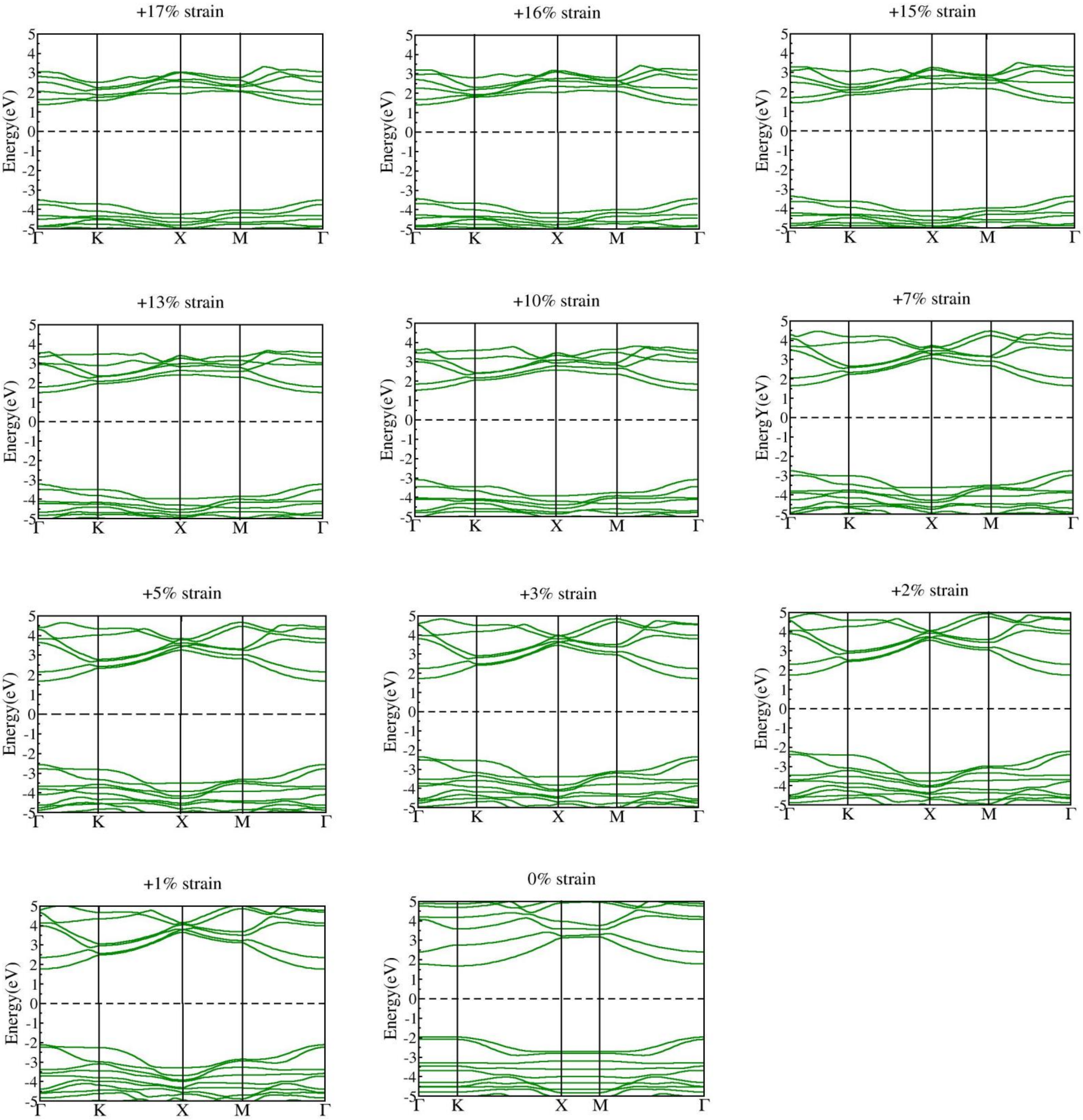

Figure S13: Electronic band structure of ψ-graphane at different values of positive strain.

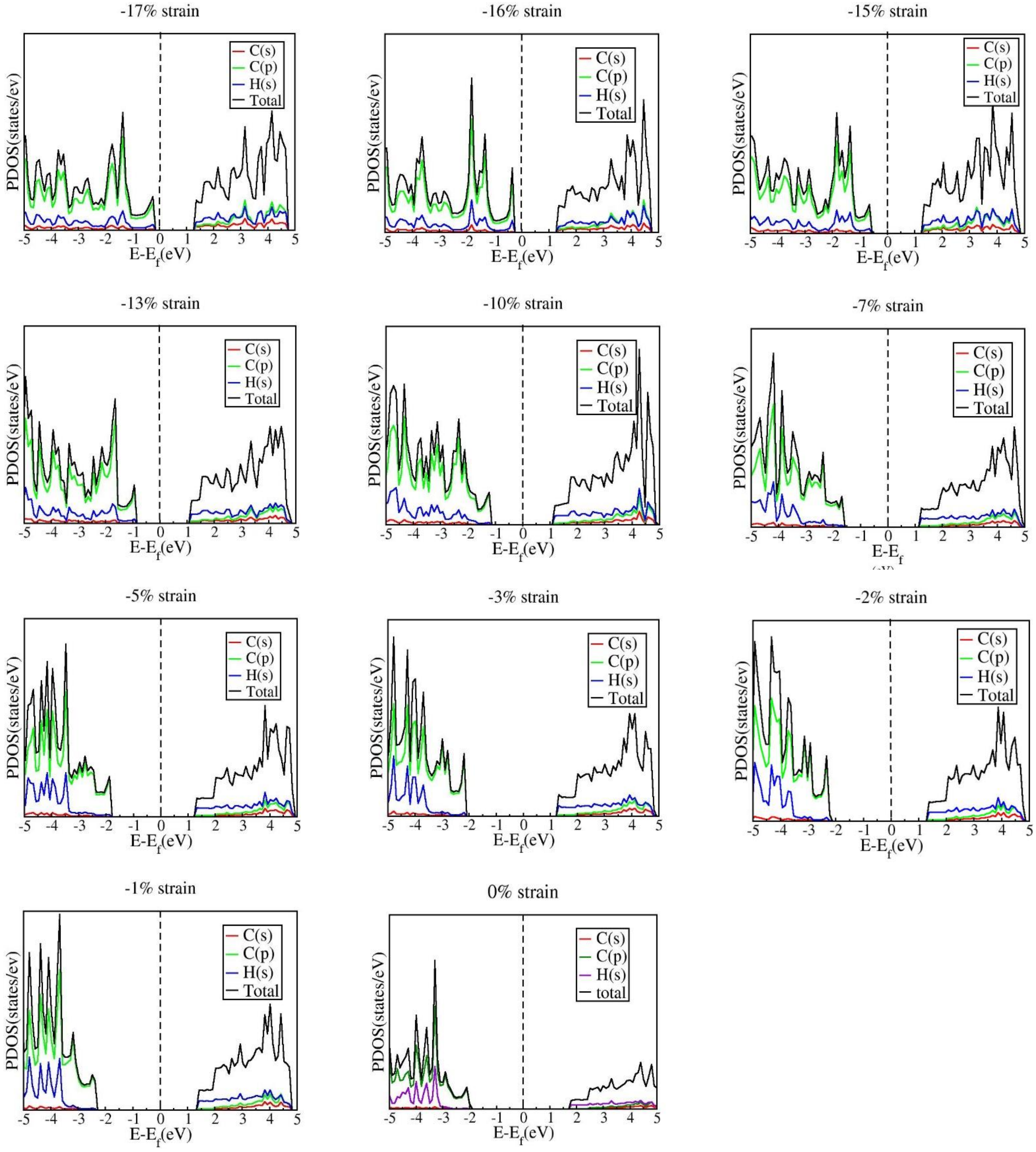

Figure S14: PDOS of ψ-graphane at different values of negative strain.

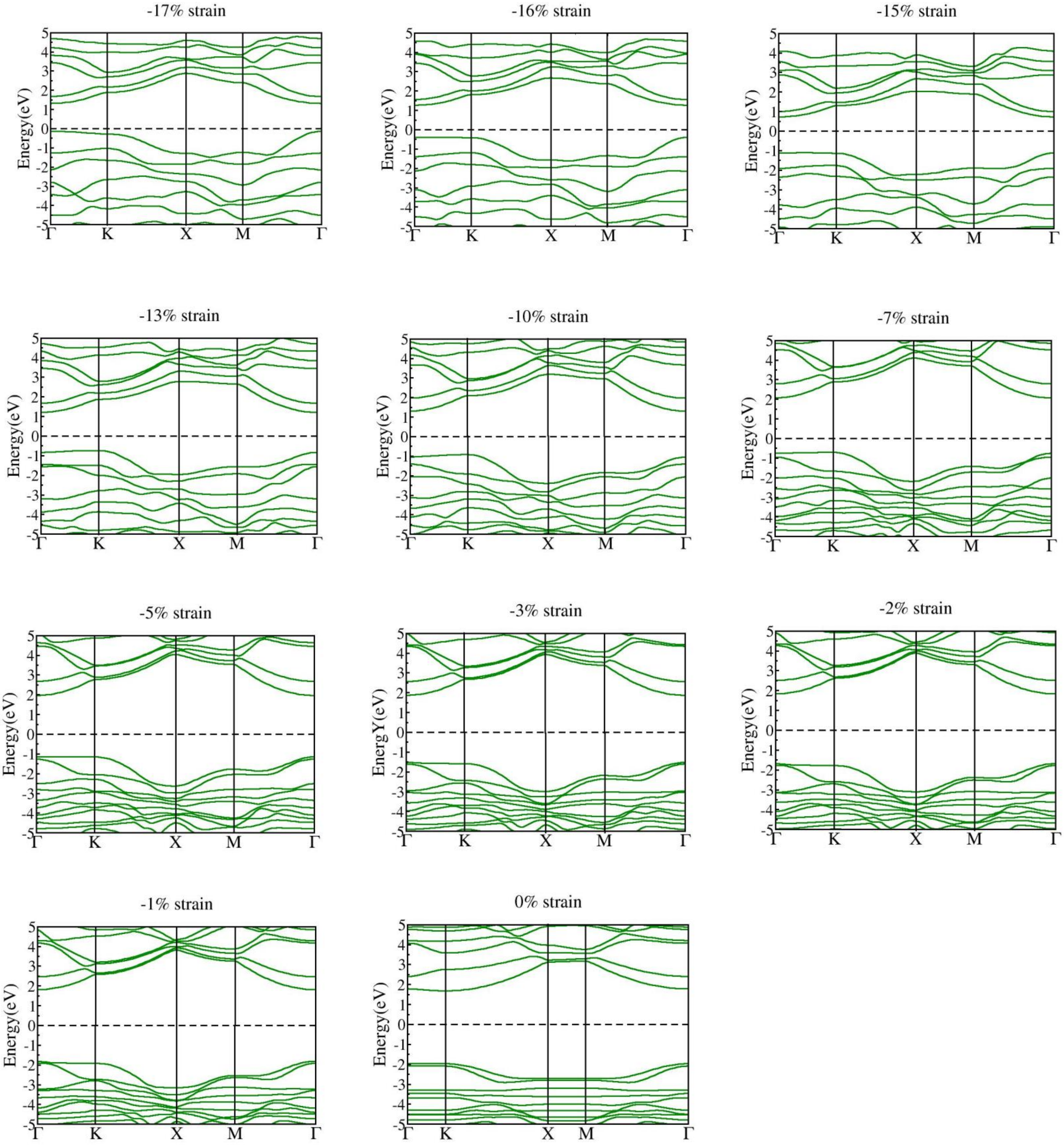

Figure S15: Electronic band structure of ψ-graphane at different values of negative strain.

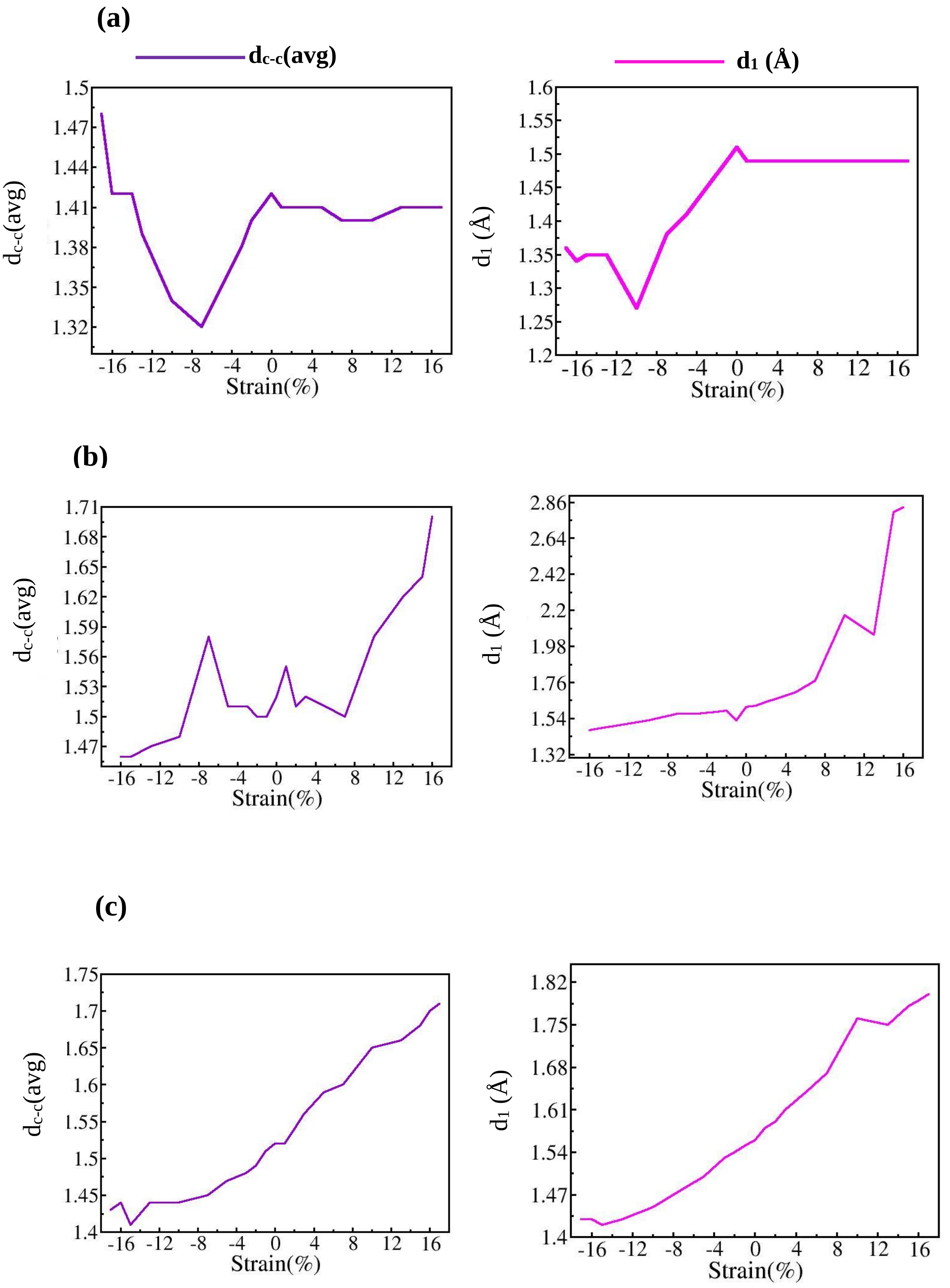

Figure S16: Variation of average bond lengths d_{c-c} (avg) (left) and d_1 (Å) (right) with applied uniform biaxial mechanical strain in (a) ψ-graphene, (b) ψ-graphone and (c) ψ-graphane.
